# Influence of Low-Level Stimulus Features, Task Dependent Factors, and Spatial Biases on Overt Visual Attention

**DOI:** 10.1371/journal.pcbi.1000791

**Published:** 2010-05-20

**Authors:** Sepp Kollmorgen, Nora Nortmann, Sylvia Schröder, Peter König

**Affiliations:** 1 Institute of Neurobiopsychology, University of Osnabrück, Osnabrück, Germany; 2 Institute of Neuroinformatics, University of Zurich and ETH Zurich, Zurich, Switzerland; University College London, United Kingdom

## Abstract

Visual attention is thought to be driven by the interplay between low-level visual features and task dependent information content of local image regions, as well as by spatial viewing biases. Though dependent on experimental paradigms and model assumptions, this idea has given rise to varying claims that either bottom-up or top-down mechanisms dominate visual attention. To contribute toward a resolution of this discussion, here we quantify the influence of these factors and their relative importance in a set of classification tasks. Our stimuli consist of individual image patches (bubbles). For each bubble we derive three measures: a measure of salience based on low-level stimulus features, a measure of salience based on the task dependent information content derived from our subjects' classification responses and a measure of salience based on spatial viewing biases. Furthermore, we measure the empirical salience of each bubble based on our subjects' measured eye gazes thus characterizing the overt visual attention each bubble receives. A multivariate linear model relates the three salience measures to overt visual attention. It reveals that all three salience measures contribute significantly. The effect of spatial viewing biases is highest and rather constant in different tasks. The contribution of task dependent information is a close runner-up. Specifically, in a standardized task of judging facial expressions it scores highly. The contribution of low-level features is, on average, somewhat lower. However, in a prototypical search task, without an available template, it makes a strong contribution on par with the two other measures. Finally, the contributions of the three factors are only slightly redundant, and the semi-partial correlation coefficients are only slightly lower than the coefficients for full correlations. These data provide evidence that all three measures make significant and independent contributions and that none can be neglected in a model of human overt visual attention.

## Author Summary

In our lifetime we make about 5 billion eye movements. Yet our knowledge about what determines where we look at is surprisingly sketchy. Some traditional approaches assume that gaze is guided by simple image properties like local contrast (low-level features). Recent arguments emphasize the influence of tasks (high-level features) and motor constraints (spatial bias). The relative importance of these factors is still a topic of debate. In this study, subjects view and classify natural scenery and faces while their eye movements are recorded. The stimuli are composed of small image patches. For each of these patches we derive a measure for low-level features and spatial bias. Utilizing the subjects' classification responses, we additionally derive a measure reflecting the information content of a patch with respect to the classification task (high-level features). We show that the effect of spatial bias is highest, that high-level features are a close runner-up, and that low-level features have, on average, a smaller influence. Remarkably, the different contributions are mostly independent. Hence, all three measures contribute to the guidance of eye movements and have to be considered in a model of human visual attention.

## Introduction

In daily life, eye movements center parts of a scene on the human fovea several times a second [1]. The part of the visual field falling onto the fovea is represented with the highest spatial acuity and, compared to the periphery, receives disproportionately more cortical processing resources [2]. The selection process is an important aspect of attention, and it has a profound impact on our perception [3]. The selection of fixation points is governed by several factors. First, goal-driven, top-down mechanisms adapt eye movements to the specific task [4], [5]. Second, bottom-up mechanisms that consider only sensory-driven aspects, such as local image features [6], contribute to the fixation selection process. Third, characteristics inherent to the visual apparatus, such as the spatial bias to the center region [7] and geometric properties of saccades [8], are widely acknowledged to influence the selection of fixation points. However, the relative roles and the interaction of these mechanisms are not understood, and a quantitative understanding of the principles of fixation selection is still lacking.

Attention models designed to cope with the complexities of natural conditions are usually based on a so-called salience map [6]. Filtering the input image with kernels reminiscent of early visual processing generates feature maps at various spatial scales. These are then combined into a single salience map, which encodes the probability that an image region will be attended [9]. In principle, the selection of features for such models is unconstrained. First implementations were designed to explain covert attention in experiments involving artificial stimuli and based on a repertoire of simple features. Present models slowly move towards a more complete list of relevant features [10] and include more and more features (Betz T, Kietzmann TC, Wilming N, König P (in press). Investigating task dependent top-down effects on overt visual attention. J Vis). Furthermore, they introduce probabilistic and decision theoretic concepts [11], [12], [13]. Salience maps predict, to some extent, fixations in complex scenes [14], [15], [16], [17], [18] for humans as well as for monkeys [19]. The critical phrase “to some extent” is at the center of an intense debate. Is it possible to refine models based on stimulus dependent salience to model overt attention as well as intersubject variability allows?

A major concern is that even if features of the salience map, such as luminance contrast, are good correlates of fixation probability, they do not necessarily drive attention causally [7], [20], [21], but are contingent on higher-order statistics [22]. These issues have raised considerable skepticism regarding models based purely on low-level image features.

For these reasons, there is consensus that viable models of human attention should not rely solely on stimulus properties. Specifically, eye movements are influenced by spatial constraints and properties of the oculomotor system. A wide range of studies has demonstrated a preponderance of small amplitude saccades [23]. Furthermore, under typical lab conditions observers have a central bias—i.e., a tendency to fixate preferentially close to the center of photographs of natural scenes, in excess of behavior under truly natural conditions [24], [25]. Furthermore, the recent years have seen a major advance in our understanding of scene layout. Including such information, which was automatically generated by machine learning algorithms, leads to a very high prediction accuracy in a search task for pedestrians [26]. Furthermore, recent work demonstrates that spatial properties might have a large influence on overt attention [27]. While it is clear that these spatial factors contribute to the selection of fixation points, there is as yet no quantification of their general influence.

That the task context influences eye movements has long been observed [1], [5]. In a study utilizing a variety of tasks—including abstract interpretations, such as the judgment of social status—the task was found to strikingly modify observed fixation patterns [5]. Also the complex activities of daily living reveal the task dependence of human eye movements [28]. Models for visual attention based solely on low-level visual features fail to capture the effects of the task context. Several extensions to existing and also new models have been proposed to address that shortcoming [29], [30], [31]. An elegant information theoretic approach combines visual appearance, spatial information and high-level information further improving prediction accuracy (Kanan CM, Cottrell GW (2010). Robust classification of objects, faces, and flowers using natural image statistics. In Proceedings of the Computer Vision and Pattern Recognition Conference (CVPR-2010)).

It was suggested early on that in a specific task context, the information content of an image patch defines its salience [32], [33]. This proposal has triggered bottom-up driven models of attention incorporating a decision theoretic approach [11], [12], [13]. Hence the information content of a patch may be viewed as a task dependent, high-level feature. This view is suited to reconciling stimulus-driven models and task-centered models. Recent studies emphasizing the importance of objects in overt attention are compatible with this view [34]. However, information content is determined either intuitively or based on a direct subjective rating. Furthermore, there is presently no general algorithm available that would reliably label objects in a visual scene. Instead studies rely again on ratings of human subjects (http://labelme.csail.mit.edu/). An explicit quantification of the contribution of task dependent factors relative to feature-based factors is still missing.

In summary, it is widely acknowledged that image features, the task of the observer, and the properties of the oculomotor system contribute to the selection of fixation points. Still, in the absence of quantitative data on the relative weight of the different factors, settling the issue of how exactly each of these contributes towards overt attention is not possible. In the present study, we attempt a step in this direction: we quantify the relative contribution of stimulus properties, task dependence, and oculomotor constraints to the selection of fixation points. We capture stimulus dependent properties by an analysis of low-level image features. Task dependent factors are captured by the information content of discrete parts of the stimulus in well defined tasks. The influence of oculomotor constraints is taken into account by a generative model including typical saccadic parameters and the central bias. With this approach we obtain scalars for each of these three factors for each image region, allowing us to quantify their independent contributions to human eye movements.

To quantify the three different types of influences we sample non-overlapping image patches (bubbles) from forest scenes and face images. These isolated patches are shown in different configurations in combination with a classification task. This design is inspired by Gosselin and Schyns, who have introduced the bubble paradigm to measure which parts of an image are used by the observer to solve a classification task [35], [36], [37], [38]. The technique applies two-dimensional Gaussian filters to isolate locations of visual cues, which are called bubbles. These are then presented in varying combinations, revealing only a limited controlled subset of the image content in combination with a classification task. Based on the observers' responses, Gosselin and Schyns derived a map revealing the regions of an image that are relevant for a specific classification task [35]. We use the bubble technique in combination with an eye-tracking experiment to obtain measures of different contributions to overt attention. Each bubble is treated as an independent unit. Utilizing recorded eye movements, responses in the classification task, feature analysis of the image patches, and baseline data taken from a free viewing eye-tracking study, we compute four measures: the stimulus dependent measure captures low-level feature contrast and is based on the luminance and texture distribution within each bubble. The task-related measure ignores image features, but quantifies how much information a bubble contains in the context of a specific classification task. Additional high-level factors, e.g. emotional and attentional state, might be relevant. We tried to keep these constant as much as possible. This quantification does of course not capture all possible top-down effects discussed in the literature as a classification task provides a particular context. The third measure, describing the spatial characteristics of eye movements, builds on a baseline study and takes into consideration the global fixation bias and geometrical properties of saccades. By evaluating the eye-tracking data of the main study, we obtain the fourth measure that captures the empirical salience of each bubble.

In comparison to full field stimuli, our bubble stimuli consist of a manageable number of discrete perceptual units. Using discrete units allows us to assign a single value for each of the measures to each bubble. In particular, describing the task dependent information of a bubble using the degree of agreement between subjects with respect to a classification task requires individual pieces of visual information. It is not clear how a measurement of local information content could be achieved using full field stimuli only. Accordingly, the problem of measuring local information is exactly the one addressed when the bubbles technique was first established [35].

Having acquired the four measures for each bubble, we finally use linear multivariate regression to quantify the overall and the individual, i.e., non-redundant, contributions of the task- dependent, feature-based, and spatial-based factors influencing attention.

## Results

In this study, subjects had to classify visual stimuli based either on face images or on forest scenes. We employed a total of four different tasks. Face stimuli had to be classified either according to ***gender*** or according to facial ***expression***, with the stimulus **classes** *happy*, *sad*, *fearful*, or *disgusted*. For the stimuli based on forest scenes, one task (***space***) was to decide whether the scenery was *close and narrow* – when the image was a close-up or displayed a closed environment – or whether it was *wide and open*. The other task was to judge the presence of indicators of human ***influence*** such as houses, roads, paths, trunks of trees. Stimulus presentations lasted for three seconds during which the subjects' eye movements were recorded. The majority of the stimuli were composed of 1 to 5 bubbles placed on a gray background. Half of the stimuli consisted of bubbles originating from the same full field image (condition ***same***), whereas 15% of the stimuli combined bubbles from different full field images belonging to the same stimulus class (condition ***congruent***). Another 15% of the stimuli were composed of bubbles originating from full field images of different classes (condition ***incongruent***). To control for position effects, we also showed stimuli (16%) in which the positions of the bubbles are shuffled (condition ***permuted***). The remaining 4% of the stimuli were full field images, which we used to confirm that subjects agreed on the classes of the images underlying the bubble stimuli. The bubbles themselves were constructed from square image patches with a side length of 6 visual degrees. To each patch, we applied a space-variant filter to imitate the retinal resolution when fixating the center of the bubble and a Gaussian mask to avoid visible edges.

75 subjects took part in this study, each performed 280 trials. We used a total of 2061 gray-scale stimuli for all subjects. This resulted in 21000 trials, recorded with 131935 fixations.

### Bubbles are Treated as Units

In a first step, we investigated viewing behavior relative to bubbles. Subjects made, on average, 6.2 fixations in each trial where bubbles were presented. Of these, 94% were no more than 3 visual degrees distant from the closest bubble center and thus were located well inside a bubble. Three percent were located at the screen center and can be attributed to anticipation of the decision screen that followed each trial. The remaining 3% were scattered across the screen. Hence, the fixations were rare in the space between bubbles and were clearly targeted at bubbles.

**Figure 1:**
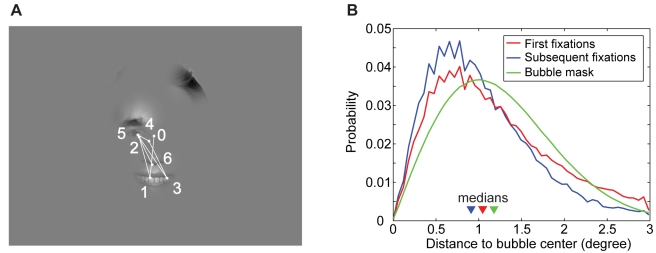
Fixations on bubbles. (A) An example trajectory recorded during the experiment. The fixation labeled with zero is the first fixation in that trial, which was excluded from analyses. (B) Distributions of distances between fixation locations and the closest bubble center for “first fixations” into a bubble (median 1.05°) and “subsequent fixations” within the same bubble (median 0.91°). For comparison, the distribution that would result if all fixations were sampled from the Gaussian window used to construct the bubbles (median 1.18°) is also given.

We designed the bubbles in such a way that maximal and complete information is available when subjects fixated the center of the bubble (see Methods). Hence subjects would not gain anything by scanning different positions within the same bubble. This, however, does not necessarily prevent them from doing so. We tested this to confirm that bubbles were indeed treated as perceptual units. Of the total number of fixations that targeted bubbles, 60% originated from outside the respective bubble (“first fixations”). The remaining 40% were due to saccades within a bubble (“subsequent fixations”). The distributions of distances to bubble centers for these two groups of fixations were significantly different (p<0.01, KS-test, Figure 1). The median of the distances to the closest bubble center was 1.05° for “first fixations” and larger than the median of 0.91° for “subsequent fixations”. For pairs of first and subsequent fixations, the subsequent fixation was, on average, 0.16° closer to the bubble center. Additionally, both distributions were more sharply peaked than the distribution that would have resulted if fixations had been sampled from the Gaussian mask used for bubble construction (p<0.01 in both cases, KS-test, see Figure 1). Altogether, the fixation data do not indicate that individual bubbles were scanned for information, but suggest that participants targeted bubble centers and made small corrective saccades towards bubble centers when landing off-center. The data is hence consistent with the assumption that bubbles were treated as perceptual units.

Building on the property that bubbles are treated as units, we derive a measure characterizing the empirical salience of a complete bubble. It is based on the fixation counts of a bubble in specific stimulus configurations (see below). In the above example of Figure 1 these fixation counts amount to 3, 3, 0, and 0 for bubbles A, B, C, and D, respectively. These counts are then averaged over subjects.

### Information of Different Bubbles is Integrated

The average classification performance for the original images (full field stimuli) was 94%, as measured by the fraction of responses that were correct with respect to the image class established in pre-experiments. For the four tasks average performance was 87%, 94%, 99% and 94% (*expression*, *gender*, *influence*, and *space*). When comparing the different tasks, please note that in *expression* the chance level is 25% and in all others 50%. The high-level of performance indicates that participants understood the tasks and had a shared interpretation of stimulus classes.

In order to be independent of predefined “correct” responses in the following analyses, we use the more general measure of *stimulus information*. It is defined by the maximal possible entropy of the distribution of responses minus the entropy of the actual response distribution (see Methods). When all subjects agree on the classification of a stimulus, that stimulus contains maximal information with respect to the classification tasks. When their response distribution is flat, the stimulus contains no information. In the case of *expression*, with 4 choices the *stimulus information* ranges from 0 to 2 bit, in the other tasks from 0 to 1 bit. *Stimulus information* thus captures the degree of consensus from the subjects classifying the stimuli.

Next we investigated stimulus information in the reduced stimuli composed of bubbles. Presenting bubble stimuli composed of bubbles taken from the same base image (condition *same*) yielded average stimulus information of 1.18 bit, 0.66 bit, 0.74 bit, and 0.54 bit for the four tasks *expression*, *gender*, *influence*, and *space*, respectively. Presenting stimuli composed of bubbles taken from different images of the same response class (condition *congruent*) average stimulus information changed to 1.31 bit, 0.62 bit, 0.67 bit, and 0.61 bit (*expression*, *gender*, *influence*, and *space*). In contrast, in presenting stimuli composed of bubbles taken from images of different response classes (condition *incongruent*) it dropped to 1.12 bit, 0.55 bit, 0.58 bit, and 0.34 bit. These data demonstrate that stimulus information is far from complete and that no ceiling effects are to be expected.

**Figure 2:**
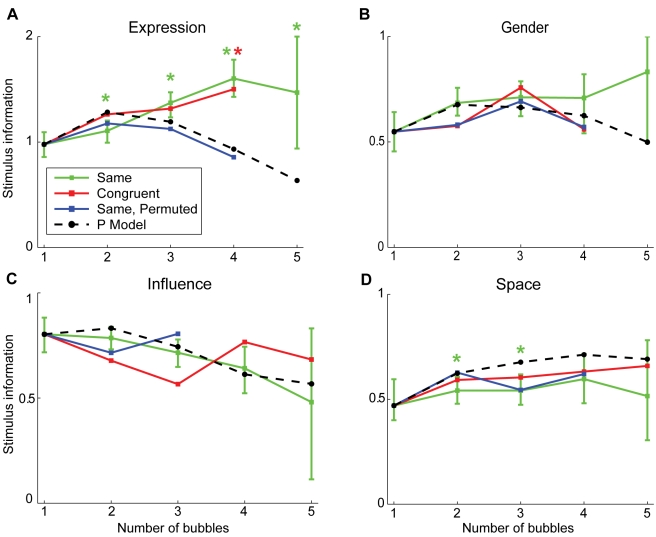
Stimulus information versus number of bubbles for the four tasks. Stimulus information estimated using the p-model is plotted for all four tasks (black dashed line). This is contrasted with the measured stimulus information in the *same* condition (green line) and in the *congruent* condition (red line). The blue line marks the measurements that result if the positions of bubble stimuli of the *same* condition are shuffled (*same*, *permuted*). The colored stars mark significant differences (p<0.05, bootstrapped confidence intervals) between the curve belonging to the respective condition and the p-model estimate. For visibility, the 95% confidence interval is marked by error bars only for condition *same*.

To address the integration of information we analyze stimulus information as a function of the number of bubbles (Figure 2). First, we compare measured stimulus information in the *same* condition with estimates of a probabilistic model of information integration (see Methods). The model, which we denote as **p-model**, integrates the response distributions of individual bubbles to estimate the stimulus response distribution and is presented here as a hypothesis. In the following, we only test plausibility of the p-model; we give a more detailed account in the Discussion. Stimulus information is computed from the entropy of the stimulus response distribution as described above. The p-model assumes independence of the information in different bubbles and integrates the information optimally. To predict stimulus information as a function of the number of bubbles, a sample of the bubbles, which were presented on their own, is selected. Then the respective response distributions of these stimuli are integrated using the p-model. This procedure is repeated 1000 times for each number of bubbles and each task. The resulting average information values are compared to the empirically found information values of the stimuli containing the respective numbers of bubbles (Figure 2). The selection of single bubble stimuli for integration is done independent of image class. In the *expression* task, which uses face stimuli, we observe a pronounced surplus of experimentally observed average stimulus information (green line) compared to the prediction of the p-model (dashed black line). This higher-than-expected stimulus information indicates a violation of the assumption of independence of the information in different bubbles and is investigated below. In the gender task, which also uses face stimuli, at four and five bubbles a surplus of measured information is observed as well. Due to the larger variance of these two data points it does not reach significance. Stimulus information in the *influence* task, which uses natural scenes, is well predicted by the p-model, and no significant deviation of estimate and data could be detected (p>0.05, bootstrapped confidence intervals). For *space*, stimulus information is a little, but significantly, smaller than predicted by the p-model (p<0.05, bootstrapped confidence intervals). In this condition, the integration strategy of the subjects does not quite reach optimal performance. These data suggest that the p-model provides a reasonable description of the information integration. The mentioned deviations are further investigated below.

Now, we investigate the integration of information for the different conditions. We compare stimulus conditions *same* and *congruent*. In contrast to the former, the latter is composed of bubbles that originate from different full field images of the same response class. Data obtained in *same* and *congruent* conditions give rise to nearly identical values of stimulus information in all tasks, and their difference is never significant (p>0.05, bootstrapped confidence intervals, Figure 2 green and red lines). Specifically, this includes the large deviation from the prediction of the p-model in the *expression* task. This indicates that the information of bubbles is integrated in the same way, irrespective of whether the bubbles originate from the *same* or different *congruent* full field stimuli.

To further elucidate the cause for the deviations of the data from the p-model estimates, we consider the interaction of bubble information and spatial location. For this purpose, we employ permuted stimuli. These are composed of bubbles placed at positions not matching their location in the respective full field stimuli (see Methods). In all tasks, including the *expression* task, the stimulus information in this *permuted* condition is well predicted by the p-model (Figure 2A, blue line). For the face stimuli, this, together with the large differences between the p-model and the *same* and the *congruent* condition for high numbers of bubbles, demonstrates that the subjects' integration of information is influenced by bubble locations. This can be understood intuitively if one assumes that bubbles at certain locations (e.g., mouth) are given more weight, irrespective of the actual content of the bubble (e.g., smile). Indeed, the main result for permuted stimuli is an improved fit by the p-model. On the other hand, position effects are not a likely cause for the deviations in the space task. There, the *permuted* and *same* conditions show no pronounced differences. The stimulus information for both is slightly below that of the p-model.

To test more directly whether bubble position and arrangement have an influence on information integration in the tasks *gender*, *influence*, and *space*, we performed an additional test and considered the differences between the response distributions of normal stimuli and their permuted versions. To specify whether these differences reflect a significant effect of permutation, we investigate whether the differences are consistent with the assumption that the responses for normal and permuted stimuli are sampled from the same stimulus answer distribution, independent of bubble arrangement. As the overwhelming majority (98.6%) of the differences between permuted and non permuted stimuli is located within the 95% confidence region of the zero hypothesis, no significant effect of permutation could be detected. It must be noted, however, that the test power is limited by the small number of trials using permuted stimuli.

We arrive at the conclusion that the p-model provides a good description of integration of information for face stimuli in the permuted condition and for forest scenes in all conditions. In the *same* and the *congruent* condition, face stimuli consisting of many bubbles are processed using additional configural information [39].

### Three Different Saliences of Bubbles and their Relation to Fixation Behavior

Now we address the relative contributions to fixation behavior of the stimulus dependent salience, task dependent information, and geometric properties of the stimuli. First, to address the stimulus dependent salience, we consider the low-level visual information of luminance contrast and texture contrast. These features are presumably processed in a bottom-up manner and have been used in other studies before. Second, to address the task dependent information, we consider the measure of bubble information, which captures the contribution of a bubble to the classification responses of subjects (see Methods). Third, to address the geometric properties, we investigate whether a simple generative model of fixation behavior that is based on the spatial arrangement of bubbles, central fixation bias, and geometrical constraints of average saccadic length and direction is informative with respect to the frequencies of fixation of different bubbles. Finally, these three components are used to explain the empirical distribution of fixations on bubbles, represented by empirical saliences. The measure of empirical salience is a context independent measure that represents how often a bubble is fixated relative to any other bubble. To obtain a measure which is independent of the specific stimulus context (instead of values for each stimulus) we combined the data from all stimuli and computed the best linear fit (see Methods). With this measure in turn the actual averaged fixation counts on the individual stimuli can be reconstructed with an average accuracy of 94.4%. Hence the empirical salience gives a faithful description of the fixation probability of a bubble in all stimulus configurations. The three former components and their relation with empirical salience are now considered in turn.

### Correlation of Low-Level Stimulus Features with Empirical Salience

In agreement with a large body of previous research [11], [12], [21], [40], [41], [42], we characterize low-level visual information contained in a bubble by its luminance and texture contrast. We estimate the contribution to fixation behavior by considering fixation probability conditioned on these feature contrasts. This allows determining the correlation of local features, as used in common stimulus-driven models of overt attention, with the empirical salience of bubbles.

The luminance and texture contrast of bubbles are determined by standard procedures (see Methods). To infer the conditional fixation probability, we recur to a previous study where gaze movements on full field images have been recorded, and the conditional probability to fixate a location given its feature values was determined empirically [43]. Here we use the same procedure and the results of the previous study to convert both luminance contrasts and texture contrasts into fixation probabilities. To obtain a model that incorporates both, we combine the resulting probabilities, assuming independence of the contributions of the two feature contrasts.

**Figure 3:**
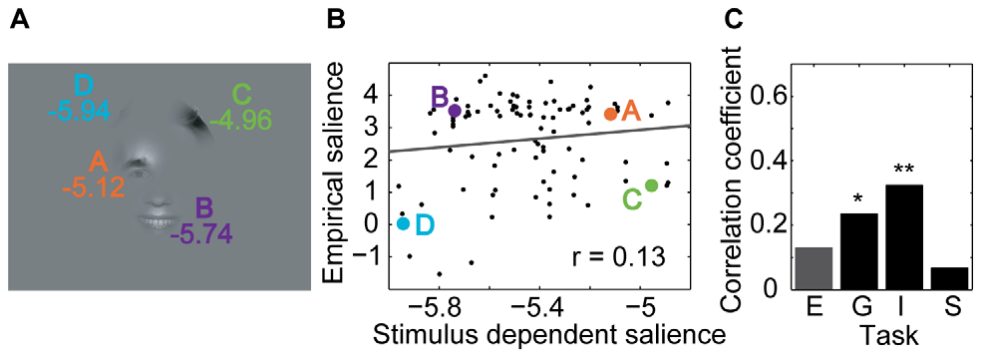
Relationship between stimulus dependent and empirical salience. (A) Example stimulus from the expression task with bubbles labeled by their stimulus dependent salience. (B) Scatter plot of stimulus dependent vs. empirical salience for the *expression* task. The positions of the bubbles from the example stimulus are marked by colored dots. The correlation coefficient r is given as a figure inset. (C) Correlation coefficients for all four tasks (E – *expression*, G – *gender*, I – *influence*, S – *space*). One star marks a significant correlation (p<0.05, t-test); two stars mark a highly significant correlation (p<0.01, t-test).

Figure 3A shows an example stimulus from the *expression* task with the individual bubbles labeled with their stimulus dependent salience. Bubble A, located on the right eye and eyebrow, contains high luminance and texture contrasts. This is mapped to a high value of the stimulus dependent salience (see Methods). Relative to the other bubbles of the *expression* task, bubble A has a high stimulus dependent salience and a high empirical salience, placing it in the upper right-hand corner of the scatter plot of stimulus dependent salience vs. empirical salience (Figure 3B). Bubble B, centered on the upper lip, has a lower stimulus dependent salience, but is looked at slightly more often than bubble A, placing it in the upper left-hand corner of the scatter plot. Bubble C, showing hair, has the strongest stimulus dependent salience of all four bubbles, but is only rarely looked at, placing it in the lower right corner of the plot. Bubble D, also showing hair, has very low stimulus dependent and empirical salience, placing it in the lower left corner of the plot. In this specific example, stimulus dependent salience and empirical salience appear unrelated.

To determine the predictive power of the feature-driven model, we correlate the predicted fixation probabilities for individual bubbles with their empirical salience (both log transformed, see Methods). Figure 3B shows a scatter plot of stimulus dependent salience of all bubbles in the *expression* task versus their empirical salience. It shows a weak, albeit not significant, correlation (p>0.05, t-test). Similarly, no significant correlation is observed for the *space* task (Figure 3C). In the remaining two tasks, *gender* and *influence*, we do observe a significant correlation. This shows that the strength of the correlation of low-level features with selected fixation points varies as a function of the task for photographs of faces as well as of natural environments.

### Correlation of Bubble Information with Fixated Bubbles

We take the contribution of a bubble to *stimulus information* as a surrogate for high-level information. We estimate bubble information for all bubbles that were shown in isolation or in combinations by assuming that the information of individual bubbles in a stimulus is integrated according to the p-model. Under this assumption, bubble information can be estimated in a global fit that maximizes the agreement between the subjects' responses to all stimuli and the alleged information contained in each single bubble (see Methods). This global fit estimates the information contained in each bubble, including those that were shown in isolation.

**Figure 4:**
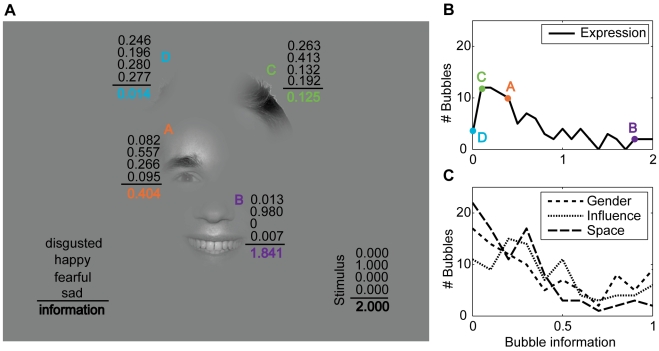
Bubble information. (A) Example stimulus from the *expression* task where the individual bubbles are labeled by their fitted response distributions and the corresponding bubble information. The four numbers above the black line give the response probabilities for the classes “disgusted,” “happy,” “fearful,” and “sad.” The bold number below the line gives the bubble information (in bit). For the whole stimulus, the measured response distribution and stimulus information (in bit) is given in the lower right corner. (B) The distribution of bubble information for the *expression* task. The bubble information of the four bubbles of the example stimulus is marked by colored dots. (C) The distribution of bubble information for the other three tasks *gender*, *influence* and *space*.

As a model of information integration we use the p-model introduced above. The results of the global fit based on the p-model may be viewed as a high-level feature specific to the context of the current task. Figure 4A shows an example of a stimulus of the *expression* task where the total measured stimulus information is 2 bit. The individual bubbles are labeled with their fitted response distributions and bubble information values. The global fit gives the information content as 0.40, 1.84, 0.13, and 0.01 bit for bubbles A, B, C, and D, respectively. In turn, estimating the stimulus information by the p-model results in 1.97 bit. This is close to the measured stimulus information with an error of 0.03 bit. Over all the bubble stimuli, the mean errors of stimulus information predicted from the fitted bubble answer distributions are 0.32, 0.20, 0.26, and 0.24 bit for the tasks *expression*, *gender*, *influence*, and *space*, respectively. For comparison, we computed the errors that would be expected if the predictions by the p-model were the true underlying response distributions of the stimuli (see Text S1 B). In that case, the subjects sample their responses from the predicted response distributions and the resulting average errors serve as lower bounds for the expected errors. The resulting errors are 0.29, 0.16, 0.16, and 0.18 bit (*expression*, *gender*, *influence* and *space*). This implies that the deviation from the p-model stays within a factor of 2 of the theoretical lower limit and is consistent with the observation above that the p-model faithfully describes the dependence of stimulus information on the number of bubbles (Figure 2). Hence, bubble information is reliably estimated by the global fit with the p-model.

Figure 4B and C show the frequencies of bubble information for the four tasks. The majority of bubbles have low bubble information values. Only a few have very high information. Bubble information varies over the whole possible range in all four tasks.

**Figure 5:**
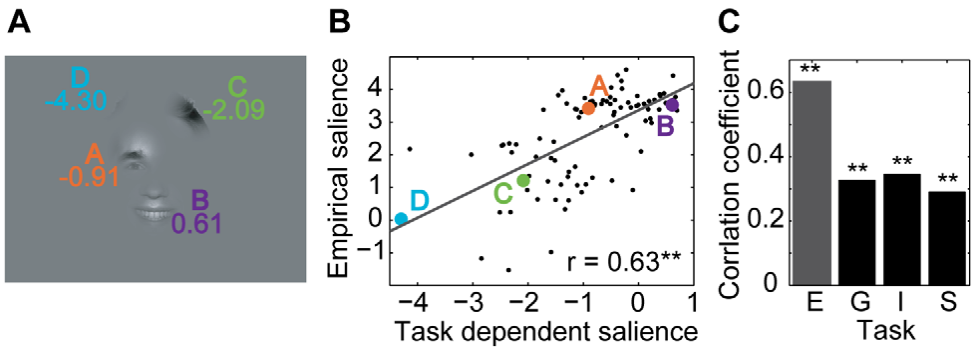
Relationship of task dependent and empirical salience. (A) Example stimulus of the *expression* task with individual bubbles labeled by their bubble information. (B) Scatter plot of bubble information and empirical salience for the *expression* task. The positions of the example bubbles are marked by colored dots. The correlation coefficient r is given as a figure inset. (C) Correlation coefficients for all four tasks. Two stars mark highly significant correlations (p<0.01, t-test).

We now investigate the correlation between bubble information and empirical salience (both log transformed, see Methods). Figure 5A shows the example stimulus with the individual bubbles labeled by their bubble information, and Figure 5B shows a scatter plot of bubble information and empirical salience for the *expression* task. Bubble A, located on the right eye, is somewhat informative and looked at very often, placing it in the upper right corner of the plot. Bubble B, located on the smiling mouth, is much more informative than A but looked at only slightly more often, placing it in the upper right corner of the plot, to the right of bubble A. Bubble C, showing hair, has less information than A and B but is still somewhat informative. It is looked at less often than A and B. Bubble D, finally, has almost no information and is also looked at very seldom. In this specific example, bubble information and empirical salience are closely related.

Investigating the complete set of bubbles, we find that for the *expression* task the correlation of bubble information and empirical salience is highly significant (p<0.01, t-test). Although there is a noticeable drop in correlation for the tasks *gender*, *influence*, and *space*; all are highly significant (p<0.01, t-test) as well (Figure 5C). Hence there are strong correlations between bubble information and empirical salience in all four tasks.

### Correlation of Spatial Arrangement with Fixated Bubbles

**Figure 6:**
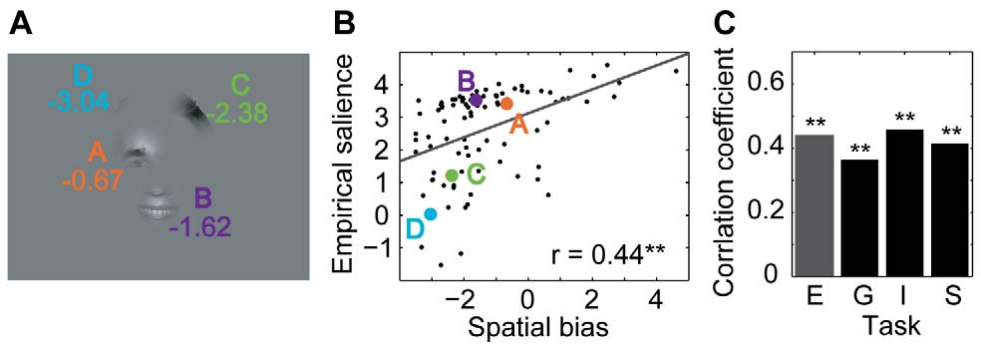
Relationship of spatial bias and empirical salience. (A) Example stimulus of the *expression* task with individual bubbles labeled by their spatial bias salience. (B) Scatter plot of spatial bias and empirical salience for the *expression* task. The positions of the example bubbles are marked by colored dots. The correlation coefficient r is given as a figure inset. (C) Correlation coefficients for all tasks. Two stars mark highly significant correlations (p<0.01, t-test).

We use a generative model to predict the empirical salience of a bubble independent of its visual content, but given its location. The generative model, as defined in the Methods section, predicts gaze trajectories on a stimulus given the initial fixation spot and the spatial arrangement of bubbles. Please note that the spatial arrangement of the bubbles alone does not contain information on the frequency of fixations on different bubbles. The model takes into account the central bias of fixations and geometric constraints on the length and direction of saccades. It does not incorporate an explicit inhibition of return (see Discussion). Both the central bias of fixations and the geometric constraints on saccades are grand averages over a large number of full field stimuli from many different categories (see Methods). The model generates fixation sequences on bubble stimuli. From these sequences the average probabilities of fixating individual bubbles on a stimulus are computed. These only locally valid values are now transformed to a global scale in the same way as the relative frequencies of fixations made by actual subjects were transformed into empirical saliences (see Methods). We now consider the correlation of this spatial bias salience with empirical salience (both log transformed). Figure 6A shows an example of a stimulus from the *expression* task where the individual bubbles are labeled with their spatial bias saliences, and Figure 6B shows a scatter plot of spatial bias salience versus empirical salience. Bubbles A and B are looked at very often and have relatively high spatial bias saliences, which is probably due to the fact that they are close to the center of the stimulus and close to each other. Bubbles C and D, which are farther away from the center and have lower spatial bias saliences, are looked at much more rarely. In this specific example, spatial bias and empirical salience are closely related.

For all bubbles of the *expression* task, the correlation between spatial bias salience and empirical salience is highly significant. For the other three tasks, the correlation is highly significant as well (Figure 6C). The correlation of empirical salience with the prediction based on spatial properties is of comparable strength in all four tasks.

### Partializing the Information in Low-Level Stimulus Features, Bubble Information, and Spatial Arrangement

**Figure 7:**
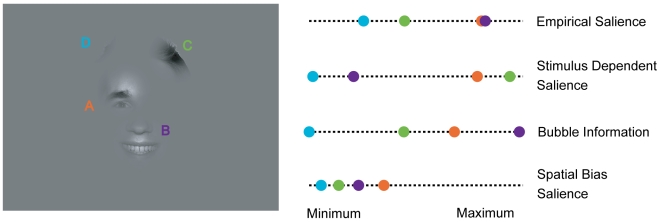
Relationship between empirical salience, stimulus dependent salience, bubble information and spatial bias salience for an example stimulus. The example stimulus from the *expression* task is given on the left. The four values characterizing each bubble are shown on their respective scales (right panel). The range of spanned values for each variable is mapped to the same interval for comparison. The colors code for the identity of the different bubbles.

For a combined view we compare the values of all three predictor variables and empirical salience for the example stimulus (Figure 7). Gathering the information from Figure 3, 5, and 6 reveals bubble information as the best predictor (e.g., the order of the bubbles according to bubble information is the same as according to empirical salience), followed by the spatial bias and the stimulus dependent salience. This example is reasonably representative for the *expression* task. In other tasks the contribution of stimulus dependent salience, bubble information, and spatial bias salience is more balanced. However, the individual correlations of empirical salience with the three predictors do not address how much the effects of one of these predictor variables are already addressed by another, because of correlations between individual predictors. In the following we address this question, which is crucial for the investigation of the causal role of the individual predictors.

We employ a multivariate linear model to predict empirical salience from the joint set of all the predictors. We analyze how well a linear combination of the stimulus dependent salience, bubble information, and spatial bias salience of each bubble can explain the attention it attracts, as reflected by the empirical salience values. As in the pair-wise correlations, we use the log transform of each predictor variable and correlate with the log transform of empirical salience. The model structure is as follows:
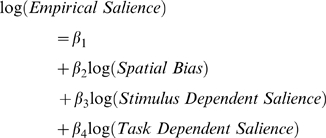
To address correlations between individual predictor variables, we use semi-partial correlations, which correlate one predictor with empirical salience while controlling for the effect of all other predictors (compare Methods).

**Figure 8:**
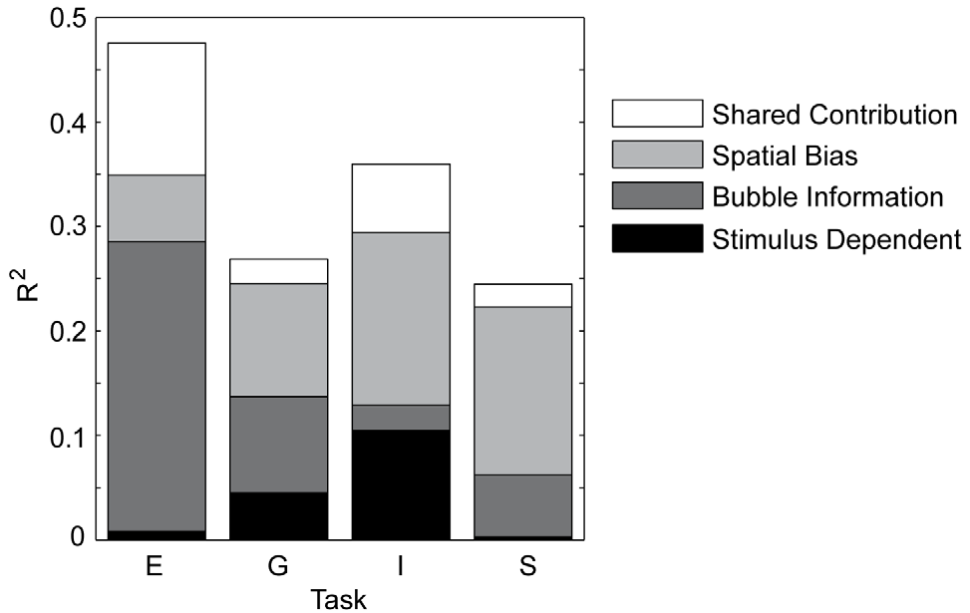
Influence of the three factors on empirical salience. The multivariate regression results are given for all four tasks *expression* (E), *gender* (G), *influence* (I), and *space* (S). The height of each bar depicts the R^2^ value; each shaded area represents the squared semi-partial correlation coefficient, which reflects the unique contribution of the respective factor. The white area in each bar represents the amount of variability of empirical salience that can explained by more than one factor.

Table 1 gives the results of this correlation analysis for the four tasks, and Figure 8 summarizes these results visually. The multivariate regression coefficient (*R*) is highly significant (p<0.01, F-test) for all four tasks, but varies considerably across tasks. For *expression*, the multivariate correlation is highly significant, with 48% of variance explained. Bubble information is the best individual predictor, the pair-wise correlation being highly significant. The individual predictive power of spatial bias salience is smaller, but the pair-wise correlation is still highly significant. Stimulus dependent salience, on the other hand, does not significantly correlate with empirical salience. These results indicate that subjects have much information about where to expect informative bubbles *a priori* and that their attention is guided by this task dependent knowledge. This is exactly what one would expect of a system that is specialized in effectively recognizing facial *expression*. It is clearly inconsistent with a purely bottom-up driven account of overt attention. For the *gender* task, the multivariate correlation coefficient is smaller than for *expression*, but still highly significant, with 27% of variance explained. Spatial bias salience and bubble information have almost the same pair-wise correlation coefficient, both correlations being highly significant. In contrast to the *expression* task, the pair-wise correlation of the stimulus dependent salience is also significant. For the *influence* task, the multivariate correlation is also highly significant, with 36% of variance explained. Again all three predictors show significant, even highly significant, pair-wise correlations. Spatial bias salience has the highest correlation coefficient, followed by bubble information and stimulus dependent salience, the latter two being almost identical. For the *space* task, the multivariate correlation coefficient is smallest, but still highly significant, with 25% of variance explained. Spatial bias salience is the best predictor, followed by bubble information. Both these pair-wise correlations are highly significant. In contrast to *influence*, the correlation coefficient of stimulus dependent salience is very small and not significant.

**Table 1:** Results of the multivariate regression.

Task		Stimulus dependent salience	Bubble Information	Spatial bias	All together
**Expression**	Correlation coefficient *r*	**0.130**	0.631**	0.437**	
	Semi-partial correlation coefficient *sr*	0.091	0.527**	0.252*	
	Multivariate regression				R^2^ = 0.476**
**Gender**	Correlation coefficient *r*	0.235*	0.326**	0.362**	
	Semi-partial correlation coefficient *sr*	0.213*	0.304**	0.329**	
	Multivariate regression				R^2^ = 0.269**
**Influence**	Correlation coefficient *r*	0.324**	0.345**	0.456**	
	Semi-partial correlation coefficient *sr*	0.324**	0.155	0.406**	
	Multivariate regression				R^2^ = 0.360**
**Space**	Correlation coefficient *r*	**0.067**	0.290**	0.412**	
	Semi-partial correlation coefficient *sr*	0.055	0.243*	0.401**	
	Multivariate regression				R^2^ = 0.245**

Pair-wise regression coefficients and semi-partial regression coefficients for the different predictors are given for each task. The total variance of empirical salience that is explained by all three factors is given in the last column. One star marks significant correlations (p<0.05); two stars mark highly significant correlations (p<0.01).

The previous observations on the relative predictive power of individual predictors in the different tasks are also supported by the semi-partial correlation analysis. The only exceptions are a rather large decrease from the pair-wise to the semi-partial correlation for bubble information in the *influence* task, reflecting a rather small unique influence of bubble information on empirical salience, as well as a noticeable decrease of the semi-partial correlation coefficient compared to the pair-wise correlation coefficient for the spatial bias based predictor in the *expression* task.

On the level of individual predictors, we make several observations. Spatial bias salience shows a strong and stable contribution in all four tasks. The unique contribution of bubble information is strong as well, but varies considerably over tasks. In the case of *influence*, it is not even significant. Stimulus dependent salience is the weakest predictor of the three, but shows significant correlations in *gender* and *influence*. Each single predictor shows significant normal and semi-partial correlations in at least some of the tasks. Furthermore, the relative contributions of the predictors, in terms of both uncontrolled and semi-partial correlations, vary considerably over tasks. Hence the contribution of the three different factors is dependent on the task, and none can be generally dismissed in an explanation for guidance of overt attention.

## Discussion

In this study, we quantify and compare the influence of low-level stimulus features, task dependent features, and spatial biases on overt visual attention. The major achievement is a direct and quantitative comparison of the individual influences of these factors on fixation behavior in a single study. The experimental approach builds on the bubble paradigm as introduced by Gosselin and Schyns [35]. It makes use of visual stimuli composed of small image patches, called bubbles, based on face images and forest scenes. Subjects classified stimuli according to facial expression and gender, or according to scenic openness and human influence, respectively. The subjects' eye movements show that bubbles are not scanned for information and verify our assumption that bubbles are treated as perceptual units. To each bubble, we assigned an empirical salience that adequately represents the fixation probability of the bubble. We further quantitatively assessed three factors that are thought to influence visual attention: first, stimulus dependent salience reflecting the probability of fixating a bubble given its luminance and texture contrast; second, bubble information reflecting how much information a bubble contains with respect to the classification task; and third, spatial bias salience reflecting the fixation probability given the location of the bubble. Bubble information was estimated based on the subjects' classification responses to stimuli composed of one or several bubbles using a model of information integration. We showed that this model is a reasonable approximation of the integration. Interestingly, we found that information of individual bubbles is integrated even if bubbles originate from different images of the same class and independent from their spatial arrangement in the case of forest scenes.

Having measured the three factors bubble information, stimulus dependent salience, and spatial bias salience, we then quantified how well they predict fixation behavior. We found that a substantial portion of variance of empirical salience could be explained by all three factors combined, although the share of variance explained varies across tasks. Pair-wise correlations between empirical salience and each of the factors indicate clear differences between the three factors. Empirical salience shows high correlations with spatial bias throughout all four tasks, whereas both the correlations with stimulus dependent salience and bubble information vary strongly with tasks. Stimulus dependent salience is the weakest predictor, but reaches significant levels in the *gender* and *influence* tasks. Bubble information is the best predictor in the *expression* task but for the other tasks it reaches slightly lower correlations with empirical salience than does spatial bias. Surprisingly, the semi-partial correlation coefficients, which reflect the unique contributions of each predictor controlling for the influences of the other factors, are only slightly lower than the pair-wise correlation coefficients. This indicates that all three factors act almost independently on visual attention. In summary, we find that all factors contribute, but that the absolute and relative strength of contribution depends on the task.

We now look into the potentially critical issues and shortcomings of our paradigm. These fall into two overall categories. First, we discuss the validity of our different measures. Second, we analyze how much the results obtained using our bubble paradigm generalize to more natural conditions.

### Validity of Bubble Measures

#### Empirical Salience

One basic assumption of the present approach is that the empirical saliences of different bubbles are independent from each other — i.e. the empirical salience of a bubble is not influenced by any other bubble on the same stimulus. An indicator of a violation of this assumption would be a change of the ratio of fixations falling onto two bubbles when other bubbles were presented simultaneously. We tested whether empirical salience values can predict the average number of fixations made by the subjects onto each bubble in all stimuli. The test resulted in very small errors showing that our assumption of independence between bubbles with respect to empirical salience is not violated.

#### Stimulus dependent salience

We characterized stimulus dependent salience as the conditional probability of fixating on an image patch given its local luminance and texture contrast for several reasons. First, these two low-level features were shown to correlate with fixation behavior in many previous studies [21], [40], [41], [42]. Hence, the present study can be compared directly with this previous work. Second, in an independent study, we observed that the strength of influence of different low-level features on overt visual attention is highly correlated over image categories and tasks (Betz T, Kietzmann TC, Wilming N, König P (in press). Investigating task dependent top-down effects on overt visual attention. J Vis). Hence, the potential benefit of additional features appears small. Third, as a control we compared stimulus dependent salience with a measure of salience obtained by a publicly available model, often used as a baseline [9], [44]. Indeed, the correlation of the two sets of saliences is high and in all tasks in the range of 0.4–0.7. Furthermore, the correlation of salience according to the model by Itti and Koch with empirical salience of bubbles is not qualitatively different from the data presented here. Fourth, previous studies showed that luminance contrast influences the response of area V1, but not the response of higher areas [45], [46]. These results indicate that luminance contrast is a good measure for the relevance of stimulus dependent signals in early visual cortex and justifies the term “low-level”. Fifth, another recent study claims that stimulus dependent salience is well described by luminance contrast without the need to introduce more complex kernels [47]. Sixth, texture contrast, which is defined as second-order luminance contrast, is usually considered a low-level feature in that sense as well and triggered some debate in the literature [40], [41]. For these reasons we decided to base our characterization of low-level contributions on luminance and texture contrast.

#### Spatial bias salience

We characterized spatial bias salience through a generative model of fixation behavior. The model takes into account the central bias of fixations (0^th^ order) and geometric constraints on the length and direction of saccades (1^st^ order). While the location of a particular fixation has an influence on the next fixation, we do not model higher order dependencies. Specifically, we do not account for inhibition of return, which would be a 2^nd^ order relation of direction and length of saccades. Inhibition of return is characterized as a small delay of saccades that return to the location of a previous fixation. As the current investigation is not concerned with these dynamic aspects, it is not of relevance here. Furthermore, recent studies report that inhibition of return might actually not change viewing strategy for complex scenes [48], [49].

#### Bubble information

Estimation of bubble information is based on the complete data set and involves a specific model of information integration. Both issues are considered in turn. In principle it would have been possible to estimate bubble information directly from stimuli presenting single bubbles only. This approach comes, however, with several disadvantages. First, the presentation of only single bubbles as stimuli is rather inefficient. To get reliable estimates of bubble information, each single bubble stimulus would have to be shown much more often. Since a participant cannot respond to the same single bubble stimulus twice and should not see individual bubbles too often, many more participants would be needed. Additionally, the responses on stimuli with several bubbles would be left unused, further diminishing efficiency. Given that in the present study 75 subjects were investigated, more than in any of the eye tracking studies cited above, this issue of efficiency quickly gets prohibitive. Second, using qualitatively different stimuli for computing empirical salience and bubble information potentially introduces systematic biases. For example, the difficulty of the classification task is increased considerably on single bubbles compared to stimuli with several bubbles. This might lead to performance near chance level, which in turn could cause subjects to lose motivation and concentration. Third, for the purpose of the present study our interest is focused on an estimate of bubble information in the context of the stimulus. In the event that estimates of information of isolated bubbles and bubbles in more complex context diverge (e.g. a systematic increase or decrease), the latter would be the relevant measure as it matches the viewing conditions during the task. These reasons further grow our confidence in the validity of the applied methods.

Several models of information integration are conceivable. The mode of information integration is an important topic in its own right and a complete treatment is beyond the scope of the present paper. We assume a probabilistic integration model but also considered two other models of information integration: first a local model that captures stimulus information by the maximally informative bubble, second a global model that differs from the probabilistic model by capturing contra factual evidence for the different choice possibilities. Compared to the p-model these models both show lower performance (see Text S1 D). Furthermore, under the assumption of the p-model being the true model of information integration, the estimates for bubble information resulting from the global fitting procedure are unbiased and have moderate variance (see Text S1 C and Figure S1). This indicates that the predictions based on the p-model are generally good estimates of bubble information.

In conclusion, although we did not show that a probabilistic model for information integration is the true or optimal model we demonstrated that the estimates for stimulus information obtained through it are robust and consistent with the majority of the data. The influence of configural information in face stimuli has been described before and does not pose a problem in the current context. The question of which is the optimal model of information integration is left to be answered by future research.

For these reasons we decided to show stimuli with varying numbers of bubbles in a homogeneous set and to employ the information integration model and global fitting procedure. In so doing we assess the two behavioral measures, bubble information and empirical salience, from the same subjects during the same experimental trials and make optimal use of experimental data to improve the signal to noise level.

#### Effects of bubble position on information integration

The discrepancy between stimulus information in bubble stimuli of condition *same* versus *permuted* in task *expression* (see Figure 2) could have several causes. The faces are similarly positioned in all stimuli so that the location of the bubbles hints at which bubbles contain relevant information: subjects might know a priori where informative regions, e.g. the eyes or the mouth, are located and select fixation targets accordingly. Furthermore, faces are special perceptual stimuli. Specific brain areas are devoted to the processing of face stimuli, and identification can be completely disrupted by reversing a face image [50], [51]. Position effects could, therefore, play a more important role for the classification of face images than for the classification of forest scenes [39]. Indeed, a major effect of permutations in the *expression* task is a largely improved fit of the p-model. This indicates that, once the standardized positioning is violated, different bubbles are treated as independent pieces of information, enabling the “normal” mode of information integration. The effect of bubble position is less pronounced in the *gender* task. For the *gender* stimuli, supposedly more regions contain information and the correlation between bubble position and bubble information is weaker. In summary, our data indicate that position effects have some influence in face stimuli, but less so in the forest scenes.

### Generalization to Full Scenes

Do the observed correlations between empirical salience, on the one side, and stimulus dependent salience, bubble information, and spatial bias salience, on the other side generalize to full field images? This is a variation of the eternal question where to place the balance between complex natural conditions and well controlled laboratory stimuli. Here, the answer depends critically on whether the four measures we employ are preserved on full field stimuli. For example, it is decisive whether the empirical salience of image patches measured on full field stimuli is comparable to the empirical salience measured on bubble stimuli. In the same way, bubble information, stimulus dependent salience and spatial bias salience need to be preserved. If the four measures that characterize a bubble were preserved when the bubble is embedded in a full field stimulus then the relationship between the measures, in particular the correlations between them, would be preserved as well and our results should generalize to full scene viewing. We consider this question for each of the measures in turn.

Stimulus based salience, as we defined it, is just dependent on a local image patch. It is thus preserved for full field stimuli. Bubble information measures how much information with respect to a task is contained within a single bubble. The amount of information contained appears largely independent of bubble context and thus only depends on the image patch itself. Spatial bias salience, as we define it, is based on global fixation and saccade biases assessed from a large variety of full field stimuli. Hence, the effect of spatial bias should be largely independent of whether an image patch is embedded into a full field or bubble stimulus. The question of whether the measure of empirical salience is preserved on full field stimuli is more intricate. The observer may very well fixate image regions in the bubble stimuli that would never draw her attention given the complete image. We tested this by correlating empirical salience of bubbles with the fixation densities of the full field images containing those bubbles (r = 0.79, r = 0.75, r = 0.55, r = 0.32 for *expression*, *gender*, *influence*, and *space*, respectively; p<0.01 in all cases). Since empirical salience of individual bubbles is well preserved on full field stimuli, we expect that our findings generalize to full scene viewing.

Previously, it was debated whether the informative regions uncovered by Gosselin and Schyns' bubble paradigm [35] are valid for full scene viewing as well. Murray and Gold argue that the bubble stimuli change the information integration strategy employed by the observer [52]. A former study showed that observers used different stimulus regions to identify faces, depending on which regions were covered by Gaussian white noise (Schwartz O, Bayer HM, Pelli DG (1998). Features, frequencies, and facial expressions [ARVO abstract #825]. Investigative Ophthalmology and Visual Science, 39(4)). It is conceivable that for full field images, which include redundant features, observers normally base their classification decision on only one or two of these features. The bubble stimuli force the observers to use different features on different trials, because only small fragments of the stimulus are shown on any given trial (Gosselin and Schyns argue, however, that these concerns are unfounded [53]). These potential problems are not relevant for our study since we do not claim that certain bubbles would be used by the observers to solve the classification task on full fields, whereas other bubbles would not. Instead, we quantify the information of each single bubble, i.e., how well the task can be solved given only this bubble. By using the information integration model, we actually incorporate the observer's strategy to use different image regions, depending on which regions are shown. Hence, our measure of task dependent information is not invalidated by the use of bubble stimuli.

In summary, we consider the present experimental paradigm a most sensible compromise, balancing between the complexities of natural conditions and well controlled laboratory stimuli, and suitable for the questions addressed.

### Relationship of Low-Level and High-Level Features to Bottom-Up and Top-Down Neural Signals

One of the most debated issues concerning overt visual attention is the role of bottom-up and top-down signals on a neural level. This issue is not integral part of the results of the current study. In the present study we discuss the influence of stimulus dependent salience and bubble information. Stimulus dependent salience translates directly to low-level stimulus features and to some degree, these features can be identified with bottom-up signals. It has been shown that neurons in V1 are sensitive to these features [45], [46], [54]. To reach relevant motor centers and influence eye movements, these signals have to traverse the hierarchy of the visual system [55]. This may be viewed as a bottom-up process. The second measure, bubble information, relates to high-level features of the visual stimulus interpreted in a specific context. Considering complex response properties in high-level brain areas, these are a natural place to extract such information [56]. Again, in view of abundant connectivity, it is plausible that such information is sent down to lower areas of the hierarchy in a top-down manner. However, receptive field properties of neurons in V1 are complex, and non-classic surround effects are far from understood [57]. Furthermore, it has been proposed that essential characteristics of a salience map are already captured in the response properties of V1 neurons [14]. For that reason we are cautious using the terms *top-down* and *bottom-up signaling*, and we took care not to make unwarranted speculations about the site of the integration of the observed contributions of low-level and high-level stimulus features.

### A Unified Theory of Overt Visual Attention

Many low-level image features were suggested to play an important role for the guidance of visual attention [9]. When compared to random image locations, fixated regions of natural and artificial images are characterized by higher decorrelation of intensities of nearby image points [42], [58], higher luminance contrast [19], [41], [42], [58], texture contrast [19], [41], color contrast [59], [60], orientation contrast [15], flicker and motion contrast [20], strong statistical dependencies between frequency components of different orientation like curved lines (Saal H, Nortmann N, Krüger N and König P (2006) Salient image regions as a guide for useful visual features. IEEE AICS), edges [18], occlusions or isolated spots [61], and disparity [62]. These effects, however, appear to be relatively weak [18], and another study reports that locations of extremes of luminance intensity, luminance contrast, high spatial frequency content, and edge density do not match with locations of fixations [63]. Yet another study puts forward contradicting evidence in favor of the role of high spatial frequency content [64]. The strength of these effects was found to vary with image type [15], [40]. Still, the idea is that with increasing complexity of the features investigated a faithful description of human overt visual attention can be reached.

This line of research has come under attack from two sides. On the one hand, Kienzle and colleagues show that much of the observed correlation of selected fixation points in a free viewing task on gray-scale images of natural scenes can be captured by an extremely simple center surround mechanism [47]. On the other hand, recent studies found that high-level features play an important role in overt visual attention and act more strongly on fixation behavior than low-level features when subjects engage in visual search tasks [65], [66], [67]. In more natural settings, task and context have a strong impact on eye movements as well [68]. Also models of visual attention that employ top-down processing were successfully applied to visual search tasks [31], [69], [70], [71], [72]. Recent work tries to combine low-level and high-level cues [73], [74]. The latter study specifically investigates the salience of light sources (very high luminance contrast) in natural scenes at dawn and dusk. They show that high-level features and spatial biases make the largest contribution in a mixture model, which is in line with the results reported here. However, in the work by Vincent et al. [74] the definition of high-level features like foreground/background contains a subjective component and might correlate strongly with low-level features like disparity. Indeed, we could recently demonstrate that disparity has a strong influence on the selection of fixation points in stereoscopic presentation [62], close regions being viewed earlier than far regions. Furthermore, about 40% of this effect survives in 2D presentation. This highlights the problem to define objectively low-level and high-level cues and to analyze their independent contribution to the guidance of gaze movements. Some experimental studies assessed the informativeness of image regions by subjective ratings [32], [33]; or they made use of identified informative regions of face images for different tasks [75]. In agreement with our data, these investigations show that fixation patterns vary for different tasks even if the visual input is identical — i.e., that high-level features like task dependent information have an influence on attention, and that more informative regions are fixated upon more often than less informative ones. The advantage of our approach is that it enables us to quantitatively measure task dependent information in an objective way. Another study presents an information theoretic approach to the combination of different cues [76]. They demonstrate that the model clearly outperforms models with pure bottom-up architectures. Furthermore, Ehinger and colleagues give a highly informative comparison with current contextual guidance models [26]. Our results are in line with these studies. Averaged over all the tasks investigated, high-level features contribute more than low-level features. Some experimental studies assessed the informativeness of image regions by subjective ratings [32], [33]; or they made use of identified informative regions of face images for different tasks [75]. In agreement with our data, these investigations show that fixation patterns vary for different tasks even if the visual input is identical — i.e., that high-level features like task dependent information have an influence on attention, and that more informative regions are fixated upon more often than less informative ones. The advantage of our approach is that it enables us to quantitatively measure task dependent information in an objective way.

One center issue of the debate about low-level and high-level features is whether, and to what degree, they have a causal role versus pure correlative effects. A study on images whose luminance contrast was locally modified shows that fixations are attracted by increases as well as decreases of luminance contrast, but that the effect within the region of normal variance of luminance contrast is small [21]. Furthermore, these observations cannot be explained by induced changes in texture contrast [40]. This argues against a causal effect, but in favor of a pure correlative effect of luminance contrast in a free viewing task on natural stimuli. Our present results agree with the aforementioned studies inasmuch as the low-level factors exhibit, on average, weak effects on fixation behavior. However, our analysis of the correlation of empirical salience with the three predictors uncovers a surprising fact. The semi-partial correlations are only a little smaller than the full correlations. This indicates little redundancy of the three predictors — i.e. low-level features are not coincident correlations of high-level features in many tasks. This argues that none of the predictors can be neglected, but that a true integration is to be achieved. This is very much in the spirit of recent proposals, putting the problem of overt attention in a Bayesian framework [11], [12].

Concerning the role of spatial biases on visual attention, it was pointed out that the spatial bias towards the screen center has to be taken into account when studying the effect of image features on selection of fixation points [7], [63]. Furthermore, some work has been done on the statistical properties of saccade length and directions. Human saccades can be modeled as a Levy flight with a heavy-tailed distribution [8] and it can be shown that under certain assumptions such a distribution leads to optimal scanning behavior. Research on higher order correlations, i.e. dependencies of selected fixation points within a trajectory, is still rare [77]. Given our current knowledge of spatial properties, a comparison of several models of fixation behavior revealed that the best performance is obtained from a strategy combining top-down information and spatial bias, which, however, was defined as the restriction of fixations to one side of the image [72]. Our results support this view, showing a surprisingly high correlation between spatial bias and visual attention. This effect is strong and consistent in all tasks tested. This contrasts with the emphasis on low-level and high-level features in current models of visual attention. Forthcoming models should put the spatial properties of eye movements on an equal footing with other factors.

The present study contributes to focusing discussions of models of attention on quantitatively testable properties. Low-level stimulus features, task dependent information content, and spatial viewing biases jointly explain a substantial fraction of the variation of empirical salience — i.e., a unifying theory of visual attention will have large predictive power. Furthermore, each of the three factors contributes significantly. A unified theory of overt visual attention has to account for all of them.

## Methods

### Ethics Statement

All subjects were informed about the experimental procedure, the eye-tracking device, and their right to withdraw from the experiment at any time. However, they were initially kept naïve as to the purpose of the experiment and were debriefed after the experiment. All participants consented in writing to take part in the experiment and to allow scientific usage of the recorded data. The experimental procedure conformed to the Declaration of Helsinki and national guidelines.

### Participants

75 student volunteers participated in the experiment (39 female, 36 male). Their ages ranged from 18 to 41, with a mean of 24.2 years. All had normal or corrected-to-normal vision, which was confirmed by a vision test with Landolt rings. Participation was voluntary, and participants either were granted extra course credits or received monetary compensation for their participation.

### Apparatus and Recording

Participants' eye movements were recorded with the head-mounted Eyelink II eye-tracking system and the Eyelink II software package (SR Research, Ltd., Mississauga, Ontario, Canada). Monocular eye-position data were sampled with infrared-based tracking only, using a sampling rate of 250 Hz. The saccade classification of the Eyelink system is based on velocity and acceleration. A saccade starts if an initial acceleration threshold of 8000°/s^2^ is exceeded and a distance of at least 0.1° is covered with a minimal velocity of 30°/s. Fixation points are then defined by the samples in between two saccades. Stimuli were presented on a 21-inch Samsung Syncmaster 1100 DF 2004 (Samsung Electronics Co. Ltd., Korea) CRT monitor at a distance of 80 cm from the subject, using a display resolution of 1024×786 pixels and a refresh rate of 120 Hz. These settings resulted in a spatial resolution of 33 pixels per degree of visual angle. No headrest was used.

### Stimuli

**Figure 9:**
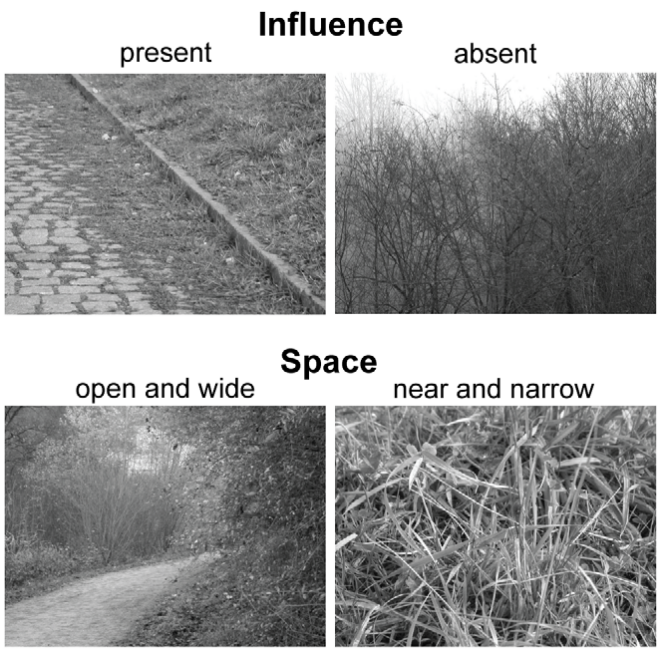
The different stimulus classes. Subjects had to classify faces and forest scenes according to four tasks (*expression*, *gender*, *influence*, and *space*). For the forest scenes, the different response possibilities are given above the example stimuli. The stimuli are shown as full fields and are used for bubble stimuli construction. For copy right reasons, we cannot show the face stimuli here but we refer the reader to Tottenham et al. [78]. The face stimuli are taken from the “NimStim” stimulus set.

All stimuli were based on gray-scale face images [78] and forest scenes (the forest scene photographs were used with permission from W. Einhäuser and P. König [21]). Photographs used for the construction of stimuli were selected on the basis of pre-experiments (forest scenes: Steinwender J (2005) Context dependency of overt attention in natural scenes. Bachelor's thesis, University of Osnabrück; faces: pre-experiment, data not shown). Face images had to be classified in different **tasks** (see below) according to gender (***gender***) and expression (***expression***), forest images according to scenic openness (***space***) and human influence (***influence***). Only photographs that were evaluated consistently by all participants of the pre-experiments were included in the present study. These responses defined the different **classes** used below during stimulus construction. We selected a total of 24 photographs of faces and 36 photographs of forest scenes. The stimulus sets were balanced in the context of each of the four tasks. In 4% of all trials, stimuli were photographs shown in full field condition (Figure 9). Although these full fields were shown during the main experiment to control for changes in classification, their main purpose was to serve as a basis for the creation of bubble stimuli. In 96% of the trials, bubble stimuli constructed from the same basic set of photographs were presented. These were created in three steps. First, 6.0° square patches were selected from the available full field photographs. Second, the image patches were space-variant filtered, imitating the retinal resolution as a function of eccentricity, and masked by a Gaussian envelope. Third, these bubbles were recombined and placed on an equiluminant gray background in different ways to create a variety of bubble stimuli. A total of 2061 gray-scale stimuli were used.

**Figure 10:**
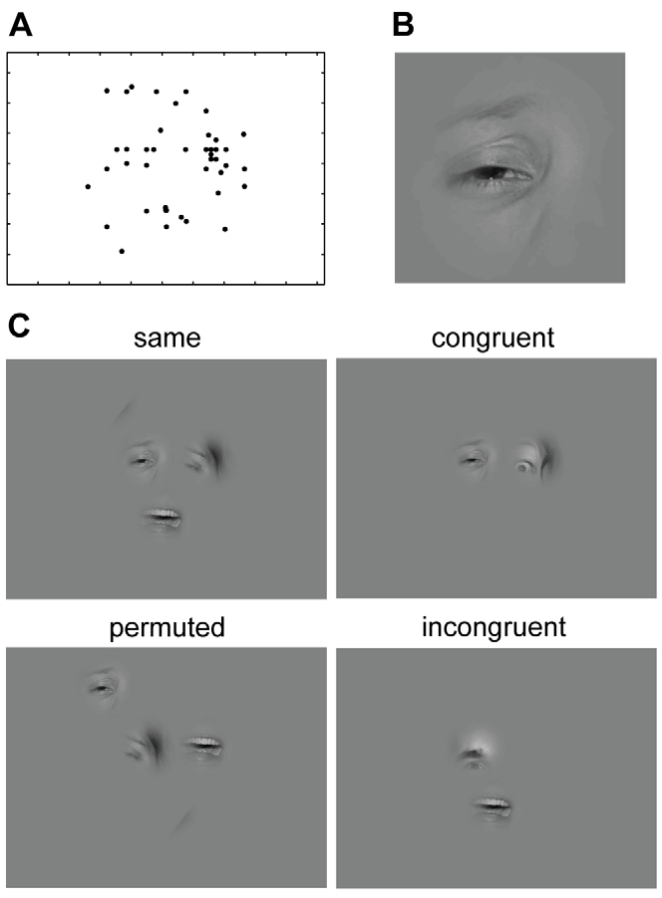
Bubble stimuli. (A) Distribution of bubble positions for the *expression* task. (B) A single bubble based on a patch of 6 visual degrees from a full field face stimulus. The patch was filtered using an eccentricity dependent frequency filter simulating the drop of spatial acuity and a Gaussian mask to avoid edge effects. (C) Different types of bubble stimuli were generated. Stimuli of the *same* condition are built from patches of the same image. Stimuli of the *congruent* and *incongruent* condition are built from patches of different images of the same class or of different classes, respectively. *Permuted* stimuli were created for each of the three conditions by shuffling the positions of bubbles.

The selection of image patches from full fields was governed by the following criteria: first, we selected image patches from locations where the fixation density obtained in the pre-experiments was very low or very high. This way of selecting patch positions yields a set of patches with diverse empirical saliences. Second, since bubbles should be independent units of information, they must not overlap. Third, for each bubble on a particular full field stimulus, there should be bubbles on other full field stimuli that occupy the same position. This constraint allowed controlling for position effects when combining bubbles from different full fields. Ideally, some of these bubbles on other full fields should be close to minima and some to maxima of their respective fixation distribution. We used a randomized algorithm to generate an appropriate selection. Since the aligned geometry of the face stimuli made it impossible to fully satisfy the latter constraint, a residual set of bubbles for the face stimuli was selected by hand. The resulting distribution of bubble centers for the *expression* task is shown in Figure 10A.

The selected image patches were first filtered using an eccentricity-dependent frequency filter that simulates the decline of visual acuity towards the edges of the visual field as resulting from the non-uniform distribution of photoreceptors on the human retina [79]. This approach ensures that all information present in a bubble can be gained by fixating the bubble center and that scanning bubbles is inefficient. To prevent potential artifacts resulting from sharp apertures, the space-variant filtered patches were masked using an isotropic Gaussian window with a standard deviation of 1.0°. This made the bubbles blend inconspicuously into the gray background. An example is shown in Figure 10B.

The final bubble stimuli were created by combining bubbles. In a small fraction, individual bubbles were shown (12%). The remaining stimuli were composed of two (42%), three (26%), four (14%) or five bubbles (2%). Combining several bubbles, depending on their full field stimulus of origin, allows different **conditions** (Figure 10C). **Same** stimuli (50% of all stimuli, including single bubbles) were composed entirely of bubbles from the same full field image. **Congruent** stimuli (15%) were composed of bubbles from different full fields that were classified in the same way during the pre-experiments (they belong to the same class). **Incongruent** stimuli (15%) were composed of bubbles from full fields of different classes. **Permutations** (16%) were created by shuffling the positions of the bubbles. The final stimulus set was created using a randomized algorithm that optimized the set with respect to the constraint that each individual bubble should appear in the same number of stimuli.

### Classification Tasks

During the experiment, participants classified visual stimuli in four different tasks. In the first task, participants tagged stimuli according to the facial *expression* of the actors into the classes “happy,” “sad,” “fearful,” or “disgusted.” Similarly they classified *gender* into “male” or “female.” For the *space* task, participants were asked to choose between “close and narrow” or “wide and open.” They were instructed to respond “close and narrow” if the image was a close-up or if it would not be possible to leave the scene—for example, if leaves and branches were blocking the view. They were told to respond “open and wide” if it was possible to look far ahead. For the *influence* task, we asked participants to look for indicators of human influence such as houses, roads and paths, trunks of trees, fences, and hewn stones and to classify the stimuli into either “present” or “absent.” The wording of the instructions was the same for all participants.

### Procedure

A complete experimental session lasted approximately one hour. It was divided into four blocks, one for each of the four classification tasks. Face stimuli and forest scene blocks were presented alternately. In the beginning of the experiment, participants were instructed about the procedure, and example bubble stimuli were shown. They were directed to classify the stimuli by pressing numbers on the keyboard's keypad and to take their best guess in cases where they were not sure about the stimulus' class.

**Figure 11:**
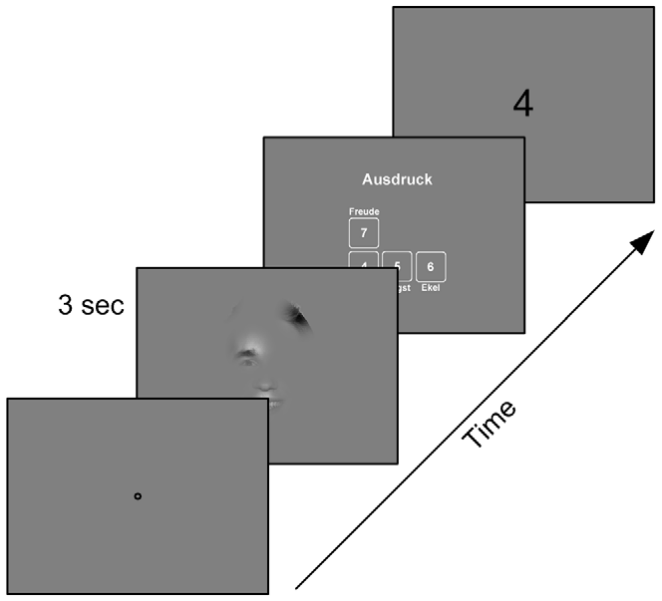
Experimental procedure. Each trial began with the presentation of a fixation point used for drift correction. Subsequently, the stimulus was presented for 3 seconds. The response screen was displayed until the subject responded to the classification task by pressing one of the indicated keys. The subject's choice was then shown as feedback.

Before the beginning of each block, the eye tracker was calibrated, and task and answer choices for that block were explained and exemplified. Each block consisted of 70 trials that were presented in constrained random order (see below). Each trial began with the presentation of a fixation cross in the middle of the screen. Whenever the fixation of the cross indicated a notable decline in tracking quality, the eye tracker was recalibrated. This ensured that the mean tracking error for at least one eye was always lower than 0.4°. If the cross was fixated properly, the conductor of the experiment triggered the stimulus presentation. We excluded the very first fixation from all subsequent analysis, as it directly reflects the preceding fixation of the fixation cross. The trial lasted for 3 seconds and was followed by the answer screen, which stayed on until participants responded by using the keyboard. There was no time limit for the decision. Before the next trial started, visual feedback of the participant's response was given to minimize classification errors due to typos (Figure 11).

The stimuli shown to each participant and their order were selected by a randomizing algorithm that respected the following constraints: for each participant, each stimulus was shown at most once; each bubble was presented at most four times; and stimuli with the same bubble were not shown in direct succession. Furthermore, on average, each stimulus should be shown to 8 participants, and the variation in the number of participants that have seen a particular stimulus should be as small as possible.

### Data Analysis

In the following, we first define a measure for the empirical salience of bubbles as quantified by fixation probability. Then we derive measures for the spatial bias, and the stimulus dependent and task dependent effects. These three measures will be used to investigate the relative contributions to the empirical salience of stimuli. All three measures put the bubbles in a global order.

#### Empirical salience

To obtain a global quantification of empirical salience, we assume that on any stimulus *S*, the ratio between the number of fixations at a bubble *A*, *F_S_(A)*, and the number of fixations at another bubble *B*, *F_S_(B)*, is independent of the context in which both are presented. This implies
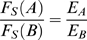
(1)for any stimulus *S* with bubbles *A* and *B*, where *E_A_* and *E_B_* are global measures of *empirical salience*, which are independent of stimulus context. From this, it follows that the equation
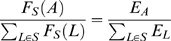
(2)holds for any stimulus *S* and any bubble *A*. Because every stimulus was presented to several subjects, we have, in fact, several left-hand sides of this equation. We average them for each stimulus and bubble. Next, the resulting equations are grouped into a linear system, and we compute the *empirical salience* as the best approximate solution. We eliminate one degree of freedom by imposing a scale, demanding that all empirical saliences sum to one.

#### Stimulus dependent salience

To characterize the bottom-up contribution to fixation behavior, we use a feature-based salience model. It models the conditional probability of fixating a location of an image, given a set of local low-level image features. Here we consider luminance contrast and texture contrast as features.

**Figure 12:**
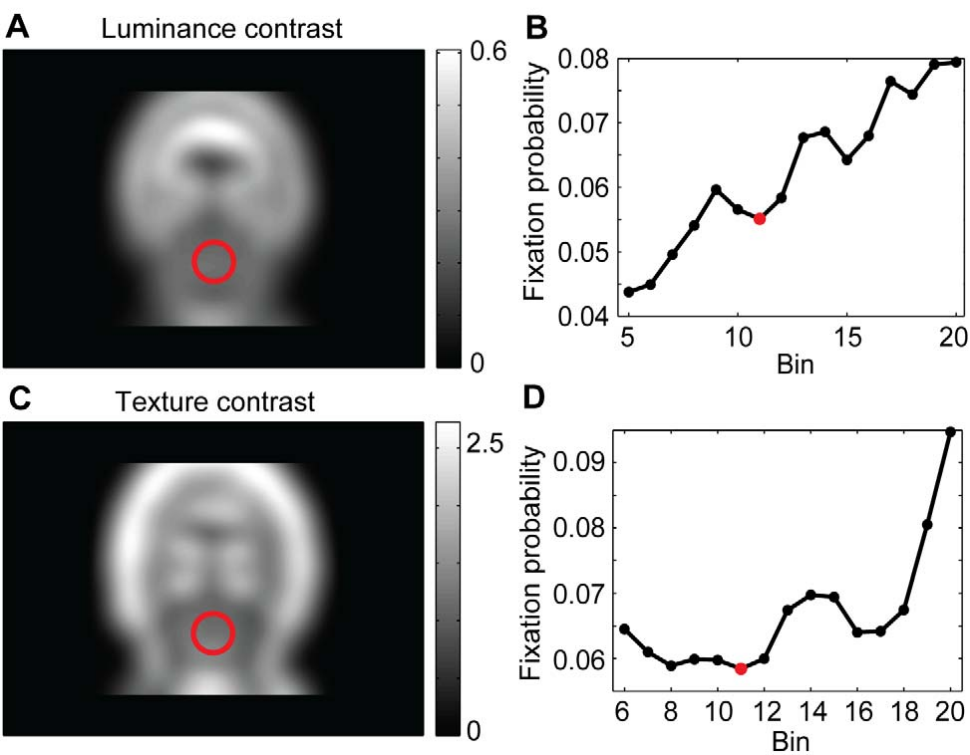
Computation of stimulus dependent salience. For each bubble, stimulus dependent salience was computed by considering the luminance and texture contrast map of the embedding full field (A and C). Luminance and texture contrast at the location of the bubble (marked by red circles for one example bubble) are then mapped to fixation probabilities (red dots). These mappings (B and D) map luminance and texture contrast bins (see text) to fixation probabilities and were obtained in a baseline study using a large number of stimuli from different categories. The resulting fixation probabilities based on luminance and texture contrast were multiplied yielding the stimulus dependent salience.

Luminance contrast is defined as the standard deviation of the luminance intensity in an image patch, normalized by the mean intensity of the entire image [19], [42]. We calculate it using circular patches weighted by a Gaussian window, *G*, in close analogy to the computation of a bubble. Formally, the luminance contrast of a pixel, *LC(x)*, is given by
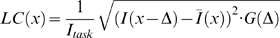
(3)where *I(x)* is the map of luminance intensity at each pixel, Δ is the displacement relative to the center of the bubble, and 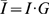 is the smooth luminance map obtained by a convolution with a Gaussian of the same size as the Gaussian used in bubble construction. Please note that the normalization deviates from the definition given by Reinagel and Zador [42] and Einhäuser et al. [19]. In these previous studies luminance contrast was normalized in each individual image. Here, however, the bubble stimuli show only a limited aperture of the respective full field stimulus. Hence varying normalization of bubble stimuli, due to not visible differences in the respective full field stimuli would make contrast values incomparable. Furthermore, in conditions *congruent* and *incongruent*, several different full field stimuli contribute. There is no obvious generalization of an image-specific normalization procedure to these conditions. For these reasons we follow the suggestion of Zhang et al. [12] and normalize luminance contrast by the mean luminance contrast over all the images of one task (*I_task_*). This is based on the assumption that the influence of a bubble's contrast on the viewing behavior depends on the whole range of contrast values appearing in the images of one category. Figure 12A shows a luminance contrast map of one of our full field stimuli.

Texture contrast is defined as the standard deviation of the luminance contrast values in an image patch, normalized by the mean luminance contrast of the entire image [19]. Formally,
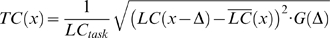
(4)where 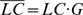 is the map of the Gaussian weighted mean luminance contrasts. Analogous to luminance contrast, we normalize by the mean luminance contrast over all images in one task. The luminance contrast map, *LC*, used for the computation of the texture contrast, is calculated with a Gaussian window of a quarter of the size of a bubble. For the subsequent computation of texture contrasts, the same Gaussian window, *G*, as for bubble creation is used. The luminance contrast and texture contrast of a single bubble are defined as the contrast values at the center of the bubble.

Based on the feature contrasts of each bubble, we now derive a scalar describing the stimulus dependent contribution to fixation probability (Figure 12). In a previous study we investigated the relation of luminance contrast and texture contrast with fixation probability in natural stimuli [40], [43]. From the observed distribution of selected fixation points and the image statistics, we used Bayes' rule to determine the conditional probability to fixate a given location. Importantly, the data were well described by a model assuming independent contributions of luminance contrast and texture contrast. Here we use this mapping, which originates from an independently obtained data set, to predict fixation probability based on the luminance contrast and texture contrast of the bubble stimuli.

For computational efficiency and optimal usage of data we bin the luminance contrast and texture contrast values of each image. We chose 20 bins with boundaries so that the number of available image locations falling into each bin is constant. Next, the probability of a feature value (luminance contrast or texture contrast) occurring at a fixated location was calculated. Then priors on the image features and fixation locations are computed. The priors on the image features are constant due to the equilibration of the distribution. The priors for the fixation locations were estimated for each image category. Both the feature and fixation location priors were corrected for the spatial viewing biases to obtain a measure based purely on low-level image features. The probability of fixating a location, given its local features, was then estimated using Bayes' rule. Finally, the stimulus dependent salience value of each bubble was calculated as the product of the fixation probabilities based on luminance and texture contrast.

#### Spatial bias salience

**Figure 13:**
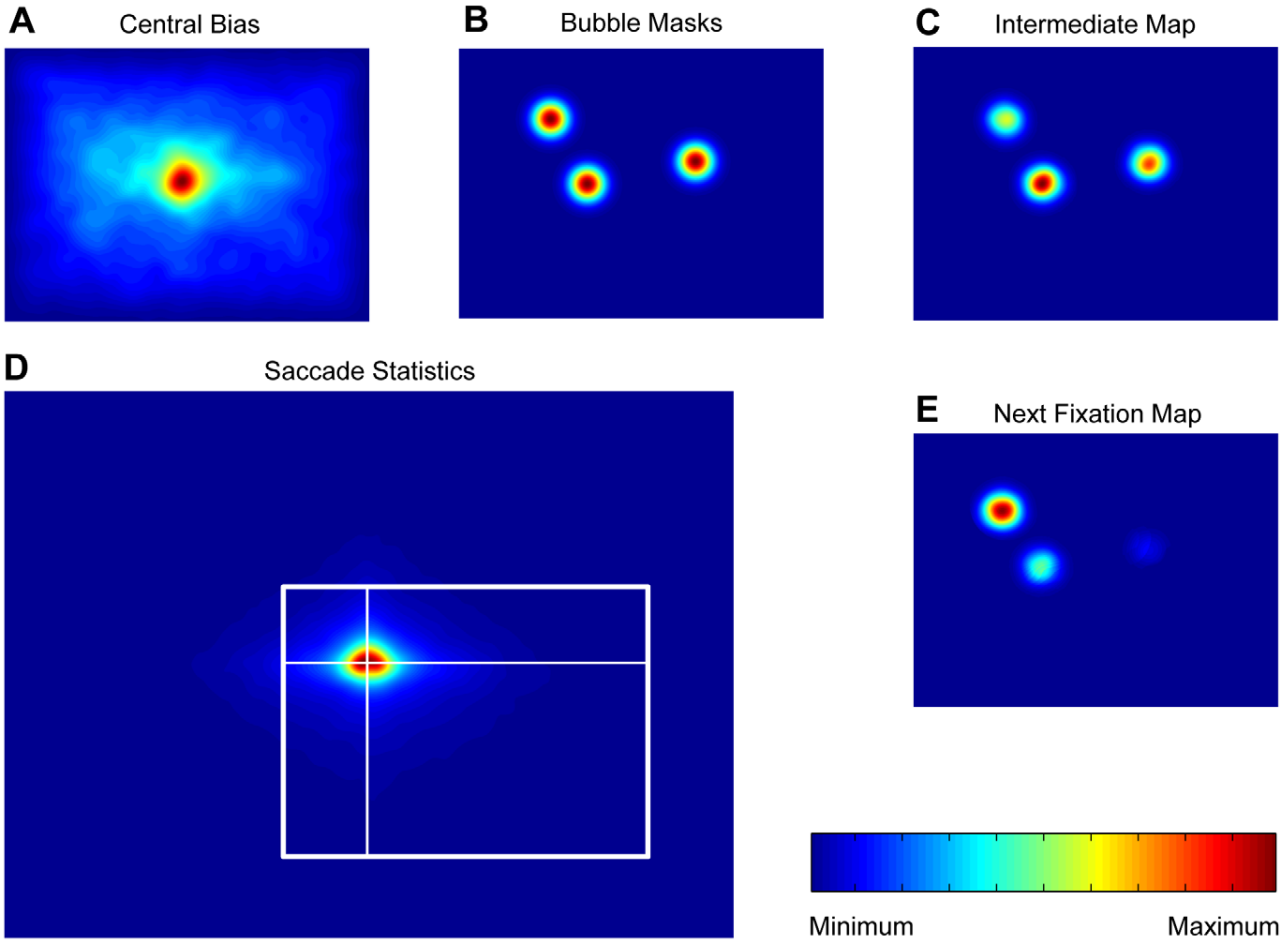
Simulation of fixation trajectories based on spatial biases. Spatial bias salience was computed from simulated fixation trajectories based on the central bias of fixations, saccade statistics, and bubble positions. Given the current fixation location, the next fixation is generated by, first, multiplying the central bias map (A) with the bubble position map (B). Second, the resulting intermediate map (C) is multiplied with the probability distribution over saccade vectors (D) centered at the current fixation. The next fixation is then sampled from the resulting map (E). For example, assuming a fixation of the upper left bubble in panel C, the multiplication (indicated by the white coordinate frame) of the intermediate map (C) and saccade statistics (D) results in the depicted next fixation map (E). Repeating this sampling procedure resulted in the simulated fixation trajectory.

As a next step we investigated to what degree the fixation of bubbles can be predicted by a spatial bias towards the screen center [7] and the statistics of saccade length and orientation [23]. Figure 13 shows the structure of a generative model based on bubble positions and on the parameters of the Gaussian window used for bubble construction (bubble masks), global fixation statistics (central bias), and saccade statistics. Using the specific bubble locations as input to the model is necessary to account for the strong fixation preference towards bubbles found in the experimental data, the very purpose of using bubbles. The fixation and saccade bias maps are derived from empirical data recorded in a previous study of our laboratory using the same experimental (Walter A (2006) Baseline Study on Overt Visual Attention. Bachelor's thesis, University of Osnabrück. Walter showed images of urban scenes/man-made objects, natural images, fractals, and pink noise images under a free viewing condition to 27 participants. We pooled over all her data from all of these categories.). The fixation bias map contains the distribution of fixations in absolute (screen) coordinates; the coordinates of fixations relative to their preceding fixations form the saccade bias map. For each trial, both maps are computed and convolved with a Gaussian kernel, with a standard deviation of 0.5° and then normalized to integral of one. Finally, we average across trials weighting each trial equally independent of the number of fixations made.

Based on the three maps, we simulate gaze trajectories of 75 virtual participants in 280 trials each in close analogy to the actual experiments. For each simulated trial, the global stimulus independent fixation map and the stimulus specific bubble mask are combined by point-wise multiplication. This combination results in an intermediate map of the spatial bias specific for the position of the bubbles in the stimulus considered (Figure 13C). Next, the saccade bias map is combined with the intermediate map by first aligning the center of the saccade map with the last fixation location (or the screen center for the first fixation within a trial), then multiplying both maps point-wise and normalizing the result to integral one (Figure 13E). The next simulated fixation is then randomly drawn from that probability distribution. This procedure is repeated until as many simulated fixations are drawn for the simulated trial as were made in the corresponding original trial.

From the simulated data we obtain a scalar measure for the fixation probability of each bubble, independent of the task and the spatial structure of the respective full field images. Instead, this spatial bias salience is based solely on the spatial position of the bubbles and the global properties of fixation points and saccades.

#### Bubble information

To characterize task dependent influences on fixation behavior, we derive a scalar measure for the information a bubble contains with respect to a classification task. First, we assume that each individual bubble is associated with a probability distribution that captures how likely the subjects are to decide for each stimulus class (*response distribution*). If this distribution is flat, the bubble contains no information relevant for classification, and performance of subjects viewing only this bubble would be at chance level. If one of its components is one and all others are zero, then the bubble contains maximal information. This is captured by the entropy of a bubble's response distribution. If *I(B)* denotes the information content and *P_R_(B)* denotes the response distribution of bubble *B*, with entropy *E(P_R_(B))*, then
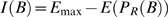
(5)where *E_max_* denotes the maximal entropy that can occur for probability distributions like *P_R_(B)* and depends only on the number of degrees of freedom of *P_R_(B)*. For tasks *expression*, *gender*, *influence*, and *space*, *E_max_* is 2, 1, 1, and 1, respectively.

Second, along the same lines we assume that our participants' responses to a stimulus *S* are independent and identically distributed according to the response distribution of the stimulus. In the case of a stimulus *S* composed of a single bubble *B* the distribution of observed answers is an estimate of *P_R_(B)*. To estimate the empirical saliences of single bubbles from measured classification responses to stimuli composed of several bubbles, we need to make an assumption on how the response distributions for single bubbles are related to the joint response distribution of a stimulus containing those bubbles. Here, we assume optimal probabilistic integration of the independent response distributions of single bubbles (**p-model**). We describe the response distribution *P_R_(S)* of a stimulus *S = {B_1_, …, B_n_}* by the function *Z* operating on the individual response distributions *P_R_(B_1_), …, P_R_(B_n_)*.
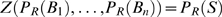
(6)We call *Z* the information integration function. It integrates the response distributions of single bubbles independent of the bubbles' absolute position or their relative arrangement. Furthermore, it does not relate to the visual content of the bubbles. It is defined as
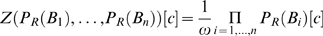
(7)where 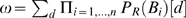, with the summation over different stimulus classes *d*, is the appropriate normalization. *Z* is formally derived by writing the probability for a stimulus *S = {B_1_, …, B_n_}* to be of class *c*, *P_R_(S)[c]*, in terms of the corresponding probabilities for the individual bubbles in *S* to be of class *c*, *P_R_(B_i_)[c]* under the assumption that the individual bubbles are independent.

For each stimulus, we can formulate an equation like (6). Hence for each task, we can formulate as many equations like (6) as there are stimuli in that task. These equations operate on response distributions. Each equation can, however, be transformed into a set of scalar equations by considering the different components of the response distributions (probabilities for the different classes) separately. This yields 1791, 600, 588, and 585 equations for the tasks *expression*, *gender*, *influence* and *space*, respectively (*expression* has four instead of two response possibilities, yielding more scalar equations). This contrasts with 282, 94, 88, and 89 free parameters in the four tasks, equaling the number of bubbles used for stimulus construction in these tasks, multiplied by the number of possible responses minus 1. We solve this over a determined system of non-linear equations by a maximum likelihood method. Details of this fitting procedure are given in Text S1 A. Finally, we determine estimated bubble information from the estimated response distributions of single bubbles according to equation (5).

#### Correlation analysis

We employ pair-wise correlation analyses (Pearson's correlations) to address the net effect of individual predictor variables. To address how well a linear combination of the stimulus dependent salience, the bubble information, and the spatial bias salience of each bubble can explain the attention it attracts, as reflected by the empirical salience values, we employ a multivariate model. Finally, to correlate one predictor with empirical salience while controlling for the effect of all other predictors, we use semi-partial correlations. For example, when we are interested in the correlation of bubble information and empirical salience controlled for the influence of stimulus-based salience and spatial bias salience, we consider the residuals of a multivariate correlation (with intersection) of stimulus-based salience and spatial bias salience with bubble information. These residuals are the differences between the prediction of the multivariate model and the actual bubble information values. We now correlate these residuals with empirical salience. The result is called the semi-partial correlation coefficient of bubble information and empirical salience.

For all, the simple pair-wise correlation analysis, the multivariate correlation and the semi-partial correlation analysis, we used the log transform of the predictor variables and the log transform of empirical salience. This standard practice [80] has the main effect of making the distributions of the individual variables more normal.

## Supporting Information

Text S1 Description of the computation of bubble information and of other models of information integration.
(0.30 MB PDF)

Figure S1 Distributions of bubble entropy estimates for different initial bubble entropies (see text) for the expression task. Each plot accumulates the data for all initial bubble entropies in a small interval around the displayed value. The distributions were obtained from simulations (see text).
(0.20 MB TIF)
