# Supplementary material for: Influence of Low-Level Stimulus Features, Task Dependent Factors, and Spatial Biases on Overt Visual Attention
Source: PLoS Comput Biol. 2010 May 20;6(5):e1000791. doi: 10.1371/journal.pcbi.1000791 (PMC2873902; doi:10.1371/journal.pcbi.1000791)
Supplement: Text S1 — Description of the computation of bubble information and of other models of information integration. (0.30 MB PDF) [file pcbi.1000791.s001.pdf]

# Influence of Low-Level Signals, Task Dependent Factors, and Spatial Biases on Overt Visual Attention

Sepp Kollmorgen[1,2+], Nora Nortmann[1+], Sylvia Schröder[1,2+*], Peter König[1]

## Supporting Text S1

### S1.A. Fitting Single Bubble Answer Distributions

Let $S$ be a stimulus made up of a number of bubbles $B_1^S, \dots, B_m^S$. If it was presented to $k$ participants, $k$ classification responses are available. By assumption (compare Methods), these $k$ responses are drawn from the same underlying stimulus response distribution. We denote that underlying response distribution by $s$. The $k$ responses yield an estimate of $s$. This estimate is a random variable following a multinomial distribution. The parameters of that distribution are given by the underlying stimulus response distribution $s$. By assumption, we know that $s$ is related to the response distributions of the individual bubbles that make up the stimulus by an information integration model $I$. Let $b_1^S, \dots, b_m^S$ denote the underlying response distributions of the individual bubbles of stimulus $S$. It holds: $s = I(b_1^S, \dots, b_m^S)$. We want to estimate $b_1^S, \dots, b_m^S$ based on the measured responses of our participants. Estimating $b_1^S, \dots, b_m^S$ is harder than estimating the underlying stimulus response distribution $s$, since we cannot directly observe realizations of the corresponding random variables. However, we can estimate $b_1^S, \dots, b_m^S$ by maximizing the likelihood of the observed classification responses with respect to the response distributions of individual bubbles. In our case, the maximum likelihood estimates of $b_1^S, \dots, b_m^S$ are those that minimize the sum-of-squares error between the classification responses predicted from them and the actual classification responses. We perform this maximum likelihood fit for every task separately, always fitting all bubbles of one task simultaneously. We used a genetic algorithm to minimize the sum-of-squares (MatLab's *ga.m* function, Mathworks, Natick, MA, USA). We used the genetic algorithm instead of gradient based methods, since we initially

experimented with a multitude of information integration models, some of them possessing non differentiable structure. The fitting procedure was repeated 15 times (each time with different random starting values for the genetic algorithm), and the best fit was chosen.

## S1.B. Comparing the Predicted Stimulus Response Distributions with the Measured Stimulus Response Distributions

We used the p-model to fit the response distributions for single bubbles. The bases for these fits are the stimulus response distributions measured throughout the experiment on stimuli with 1 to 5 bubbles. As an additional test for the validity of the p-model we predicted the stimulus response distributions from the fitted response distributions for single bubbles using the p-model. We give the absolute difference of stimulus information of the predicted response distribution and stimulus information of the measured response distribution averaged over all stimuli within one task to indicate the fitting error. As a lower bound, we compute the error that would be expected if the predicted stimulus response distributions were the true response distributions. Under this assumption, the measured response distributions are built up from samples drawn from the predicted response distributions. Hence, we computed the average absolute difference between stimulus information of the "resampled" response distribution and the assumed response distribution.

## S1.C. Validating the Fit

Because of the random nature of the fitting algorithm and the measured stimulus answer distributions, we investigated the quality of the estimation process. We investigated the bias and variance of the estimated underlying bubble distributions using simulations of the experimental process and the fitting procedure, based on random initial values for the underlying bubble distributions (these values were, however, chosen to be close to the estimated underlying bubble answer distributions to achieve maximal comparability). The simulation proceeded as follows. First, we choose underlying response distributions for single bubbles. Second, we use the integration model to compute the response

distributions for whole stimuli. Third, we resample those distributions, taking into account the number of subjects that actually saw each of the stimuli in the real experiment. Fourth, we perform the above described fit to obtain estimates of the underlying response distributions for single bubbles based on the resampled responses to whole stimuli.

These four steps are repeated 30 times, yielding pairs of underlying bubble response distributions and their estimates for every repetition. We are interested in the bias and variance of the entropy of the estimated response distributions, because the entropy is what we correlate with empirical salience and our other measures. Hence, for each pair of the underlying "true" bubble response distribution and its estimate, we compare the entropies of the distributions. Figure S1 shows how the entropies of the estimated bubble response distributions relate to the entropy of the true response distributions for different values of the true entropy for the *expression* task. The estimates appear unbiased, and the variance is moderate. The situation for the other tasks is qualitatively the same.

### S1.D. Other Models of Information Integration

We assume a probabilistic integration model but also considered two other models of information integration. First, a local model captures stimulus information by the maximally informative bubble (max-model). This model always selects the bubble with the highest information. Formally:

$$Z_{Max}(P_R(B_1), \dots, P_R(B_n)) = \mathrm{P_R}(\operatorname*{argmax}_{B_i, i=1..n} I(B_i))$$

Where $P_R$ denotes the response distributions, $B_i$ denote an individual bubble and $I(B_i)$ denotes its information content. $Z_{Max}$ is a function on the response distributions of the individual bubbles (just like in the case of the p-model) and its value is the response distribution with maximal information.

Second, we considered a global model that differs from the probabilistic model by capturing contra factual evidence for the different choice possibilities (ce-model). Formally:

$$Z_c(P_R(B_1),\ldots,P_R(B_n))[c] = \frac{1}{\omega}(1 - \prod_{i=1,\ldots,n}(1 - P_R(B_i)[c]))$$

We evaluate these models using the method employed to generate Figure 2 (see Results). We give the squared error between the same condition and the model prediction (divided by the maximal information of the task) averaged over bubble numbers and tasks. The average squared relative error for the max-model is 0.044276. For the ce-model the average error is 0.23654. The error for the p-model is 0.02518.
