# Supplementary figures and images for: Influence of Low-Level Stimulus Features, Task Dependent Factors, and Spatial Biases on Overt Visual Attention
Source: PLoS Comput Biol. 2010 May 20;6(5):e1000791. doi: 10.1371/journal.pcbi.1000791 (PMC2873902; doi:10.1371/journal.pcbi.1000791)

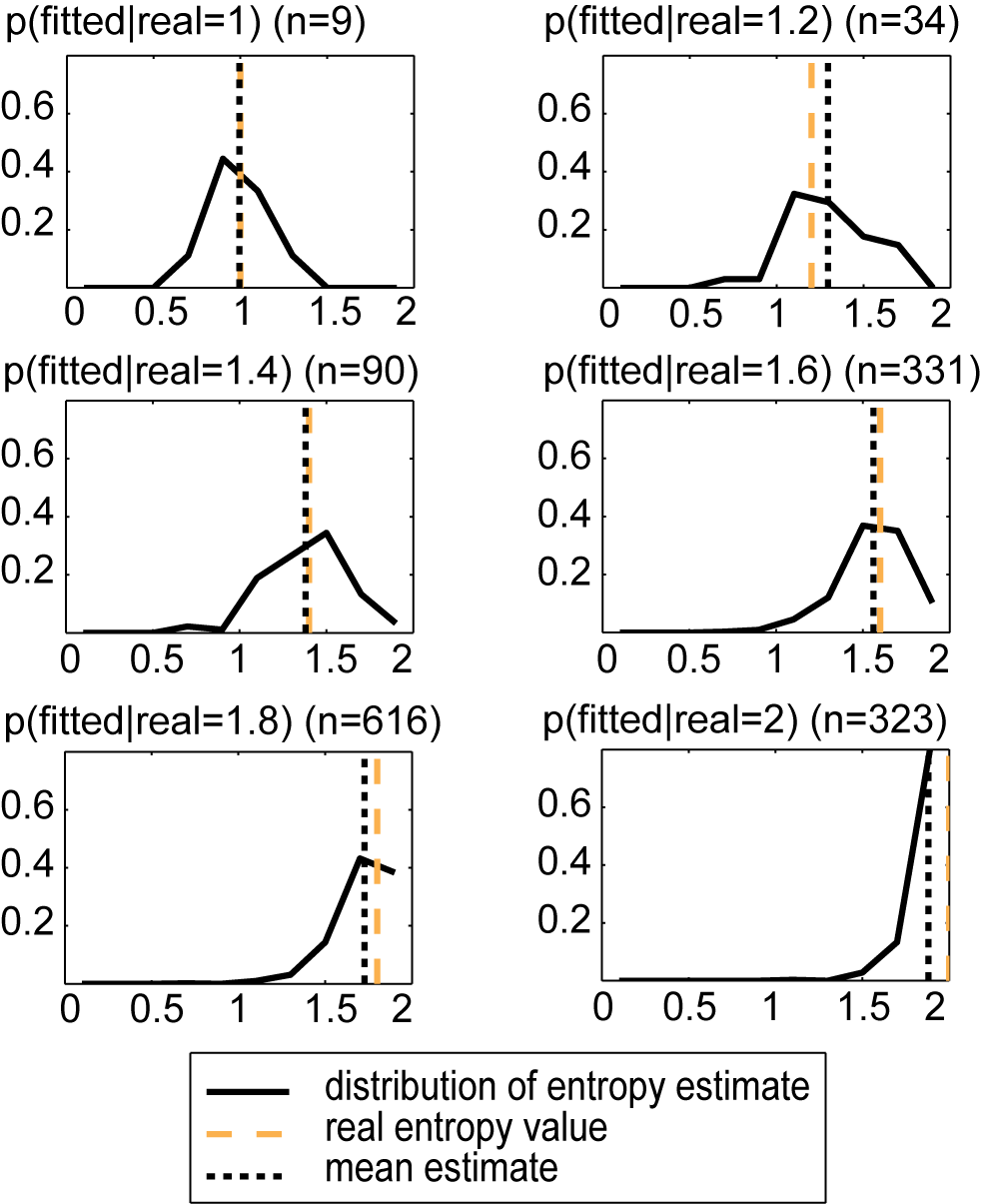

Supplement: Figure S1 — Distributions of bubble entropy estimates for different initial bubble entropies (see text) for the expression task. Each plot accumulates the data for all initial bubble entropies in a small interval around the displayed value. The distributions were obtained from simulations (see text). (0.20 MB TIF) [file pcbi.1000791.s002.tif]
